# Invasive Strawberry Guava weakens avian frugivory in native forests of an oceanic island

**DOI:** 10.7717/peerj.21749

**Published:** 2026-09-30

**Authors:** Ghanishta Seeburrun, Cláudia Baider, François Benjamin Vincent Florens

**Affiliations:** 1 Tropical Island Biodiversity, Ecology and Conservation Pole of Research, Department of Biosciences and Ocean Studies, Faculty of Science, University of Mauritius, Le Réduit, Mauritius; 2 The Mauritius Herbarium, Agricultural Services, Ministry of Agro-Industry, Food Security, Blue Economy and Fisheries, Le Réduit, Mauritius

**Keywords:** Artificial fruit, Ecological restoration, Frugivorous birds, Invasive alien plants, Mauritius, Passive acoustic monitoring, *Psidium cattleyanum*, Seed dispersal, Weed control

## Abstract

Frugivorous birds play a vital role in tropical forests by dispersing seeds and facilitating plant regeneration. The effectiveness of frugivores, including birds, as seed dispersers depends in part on their fruit removal rates. Although invasive alien plants (IAP) may influence the seed-dispersal role of birds in varied ways, few comparative field studies have dealt with avian frugivore-plant interactions, particularly in settings of advanced invasion on oceanic islands. We assess how IAP influences birds’ attack rates on fruit models, to explore the ecological and conservation implications for avian seed dispersers and the plants that they disperse. We studied and compared two protected native forests on the oceanic island of Mauritius (Mascarenes), each comprising an area weeded of IAP (since 1990s) and an adjacent area invaded primarily by Strawberry Guava (*Psidium cattleyanum*), the overwhelmingly dominant invasive woody plant in Mauritian lowland wet forests. We quantified avian frugivory in weeded and invaded forests using artificial fruit models made from non-toxic modelling clay, which were collected 72 h later to quantify avian feeding attempts (based on beak marks on the models). Moreover, passive acoustic monitoring was used to assess frugivorous bird assemblages and overall activity across forest types. This procedure was repeated 11 times over 1 year. Bird species richness was comparable across the two forest types, but total acoustic detection rate was higher in the weeded forest at both sites, with a moderate shift in bird assemblages in the invaded forests. Native bird species tend to be most negatively impacted by IAP. Avian attack rates on fruit models were about thrice higher in weeded than in invaded forests at both sites, and were positively associated with increased Acoustic Complexity Index (ACI) values, suggesting greater bird activity and foraging effort in weeded forest. Invasion by the dominant fleshy-fruited IAP reduces habitat quality for native frugivorous birds and weakens bird–plant interactions, potentially disrupting bird-mediated seed dispersal. This may be driven by two mechanisms: native fleshy-fruited plant species produce fewer fruits in invaded forests and/or forest penetrability by birds may be lowered by an extremely high stem density of understorey weeds. Conservation strategies should prioritise *in situ* control of IAP to reverse the lowered carrying capacity of birds, particularly given that only 8% of surviving natural habitats have been weeded, while the remaining 92% continue to be degraded by IAP. Weeding larger native habitat areas would promote natural regeneration of native plants, while fostering the recovery of threatened native bird species. Over time, this would enhance ecosystem resilience and reduce support for currently intensive and costly management interventions like planting, which largely constrain areas that can be managed for conservation.

## Introduction

Frugivory is a vital mutualistic interaction in which plants produce nutrient-rich fruits consumed by frugivores, that subsequently facilitate seed dispersal, thereby contributing to the natural regeneration of plant populations and the maintenance of the community structure (Jordano, 2000; Şekercioğlu, Daily & Ehrlich, 2004; Jordano et al., 2007; Muñoz et al., 2017). It is estimated that 25–80% of temperate plant species and 40–90% of tropical rainforest woody species rely on frugivory by vertebrates for seed dispersal (Howe & Smallwood, 1982; Jordano, 2000). Birds are among the most important vertebrate frugivores (Fleming & Kress, 2011), and act as primary seed dispersers of many plant species (Bascompte & Jordano, 2007; Arruda, Rodrigues & Izzo, 2008; Schupp, Jordano & Gómez, 2010; Kuhlmann & Ribeiro, 2016). Their effectiveness as seed dispersers depend on both quantitative and qualitative components of seed dispersal effectiveness (Jordano & Schupp, 2000; Schupp, Jordano & Gómez, 2010; Corlett, 2017).

The quantitative dimension, including visitation frequency and the number of fruits handled per visit, are strongly influenced by frugivore foraging behaviour (Vázquez, Morris & Jordano, 2005; Schupp, Jordano & Gómez, 2010; Ferger et al., 2016). During foraging, birds respond not only to the intrinsic qualities of the fruit, such as colour, odour, size and nutritional content (Herrera, 2002; Galetti, Alves-Costa & Cazetta, 2003; Gagetti, Piratelli & Piña-Rodrigues, 2016; Hazell et al., 2023), but also to the spatial context of fruiting plants (Martin, 1985; Fontúrbel et al., 2015; Cazetta et al., 2019). For instance, studies have shown that frugivorous birds preferentially forage in habitats with greater fruit abundance, density, and species richness, where search effort is reduced and energetic returns are maximised (Saracco et al., 2005; Kissling, Rahbek & Böhning-Gaese, 2007; Carlo & Morales, 2008; Smith & McWilliams, 2014).

However, invasive alien plants (IAP) are recognised as one of the major threats to frugivory, especially on islands (Traveset & Richardson, 2006; McConkey et al., 2012; Early et al., 2016; Rumlerová et al., 2016). They can outcompete native plants (Vilà & Weiner, 2004), resulting in modification to forest structure, vegetation composition (Aravind et al., 2006), and resource availability (Morales & Traveset, 2009). These changes can subsequently influence the distribution and behavioural patterns of avian frugivores (Aravind et al., 2010; Grzędzicka & Reif, 2020; Da Silva et al., 2020; Stewart et al., 2021), which may lead to decreased visitation and reduced consumption of fruits (Schaefer & Ruxton, 2011; Heleno, Ramos & Memmott, 2013; Costa et al., 2022b).

Conversely, IAP can also exert facilitation effects on frugivory. By serving as a food resource, providing structural habitat, or attracting generalist frugivores, they may enhance visitation rates in invaded habitats and, under certain conditions, promote seed dispersal and forest regeneration (Erskine Ogden & Rejmánek, 2005; Neilan et al., 2006; Berens et al., 2008; Gleditsch & Carlo, 2011; Jacob, Lechowicz & Chapman, 2017; Gomes et al., 2021; Vergara-Tabares et al., 2021). Given that the impacts of IAP on frugivory vary across ecological contexts, with both positive and negative effects documented (Heleno, 2020; Sperry et al., 2021; Costa et al., 2022a, 2022b; Cordero, Gálvez & Fontúrbel, 2023; Dáttilo, Luna & Villegas-Patraca, 2023; Marciniak et al., 2024), specific research is warranted in areas where such impacts have not yet been investigated, so as to better inform management and restoration strategies. This is particularly important in oceanic island systems, which are highly vulnerable to biological invasions and face an elevated risk of disruption to plant–animal mutualisms (Aslan et al., 2013; Díaz et al., 2013; Dawson et al., 2017; Heinen et al., 2023, 2025; Llorente-Culebras et al., 2024).

The oceanic island of Mauritius is ideal to investigate the effect of IAP on avian frugivory. Since human colonisation in 1638, habitat destruction spared only 4.4% of its original native vegetation (Hammond et al., 2015), much of which is heavily invaded by IAP (Florens et al., 2016). The high historical rates of vertebrate extinction in Mauritius have resulted in a vertebrate frugivorous community composed of merely a few native and many alien species, including birds, mammals and reptiles, but these do not compensate for the ecological roles of extinct frugivores (Heinen et al., 2023). Many alien frugivores act as seed predators rather than effective dispersers, are unable to swallow and disperse large seeds handled by native species, or may facilitate dispersal of invasive plants (Baider & Florens, 2006; Cheke & Hume, 2008; Linnebjerg, Hansen & Olesen, 2009; Reinegger et al., 2021; Heinen et al., 2023). Such loss of frugivores has been documented to result in weaker regeneration in certain native fleshy-fruited species in the Mascarenes (Albert et al., 2021).

Past research has mainly focused either on threatened bird species (Cheke, 1987; Jones, 2004), or more recently, on the structural change of native forests driven by the ongoing progression of IAP, leading to a decrease in species richness (Baider & Florens, 2011), density, and biomass of native trees (Florens et al., 2017b). These changes are attributed to an increase in native tree mortality (Baider & Florens, 2006; Florens et al., 2017b), coupled with a decrease in growth rate (Florens, 2008), and reduced flowering, fruiting (Monty, Florens & Baider, 2013; Krivek et al., 2020) and regeneration (Florens, 2008; Baider & Florens, 2011). However, experimental comparisons examining how woody IAP influence avian foraging are rare (Heleno et al., 2010; Marciniak et al., 2024).

This study investigates how IAP impacts frugivory by birds. Our objectives were to: (i) compare avian frugivory between forests invaded by IAP and forests undergoing restoration following the removal of IAP by comparing frequency of beak marks left on fruit models (we could not use natural fruits because their abundance in the two forest types are inherently different, with more fruits present in the weeded areas) (Baider & Florens, 2006; Monty, Florens & Baider, 2013; Krivek et al., 2020), (ii) assess differences in frugivorous bird assemblages between these contrasting forest types, and (iii) analyse how overall bird activity reflect frugivory intensity across habitats. We hypothesise that the abundance of frugivorous birds would be higher in weeded forests than in invaded forests, as the removal of IAP is expected to improve habitat quality and increase the availability and accessibility of native food resources. Consequently, we predict that the encounter rate between birds and fruit models, and therefore, the frequency of beak marks reflecting frugivory intensity, would also be higher in weeded forests compared to invaded forests. Finally, we predict that frugivory rates would be positively associated with avian activity levels measured using acoustic indices, reflecting greater encounter rates between birds and artificial fruit models.

## Methodology

### Study area

The study was carried out at the only two suitable sites for such a study on the island, namely at Brise Fer (BF) and Mare Longue (ML), located within the Black River Gorges National Park (BRGNP) and separated by approximately 2 km (Figs. 1A and 1B). These two sites are representative samples of the dominant vegetation type of Mauritius (Florens et al., 2012) and were the only places where large enough weeded areas (at least 2 ha) were created in the 1990s that allowed for the study of frugivory within weeded areas while minimising the potential edge effects arising from their juxtaposition with non-weeded forest around them. Similarities between the two sites include vegetation type and structure, bird community, and conservation management actions for nearly three decades. These selected sites also have a large enough area to allow the placement of the clay models in different sections and on individuals in different months.

**Figure 1:**
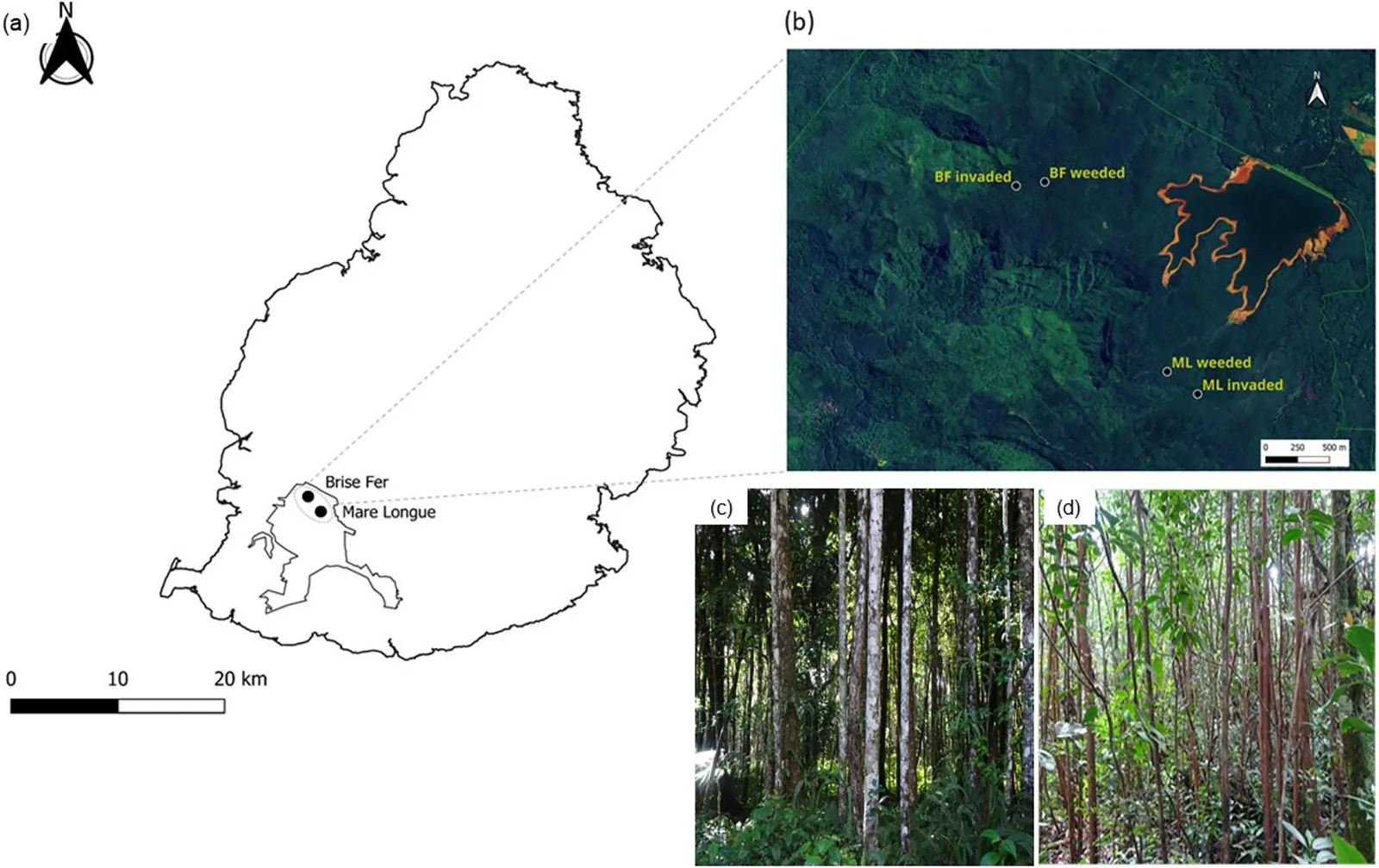
Location of the study sites within the Black River Gorges National Park (BRGNP), Mauritius. (A) Map of Mauritius showing the position of BRGNP and the study sites of Brise Fer (BF) and Mare Longue (ML). The Mauritius outline was digitised by the authors from © 2026 Google Earth Pro imagery; (B) Zoomed view of the study area indicating a center point of invaded and weeded forest plots. Plot locations were recorded using GPS during field surveys and mapped in QGIS version 3.34 using background satellite imagery © 2026 Google Earth Pro; (C) Forest where alien plants have been cleared; (D) Forest invaded by alien plants, the reddish brown stems are that of the invasive Strawberry Guava (*Psidium cattleyanum*). Photo credit: Ghanishta Seeburrun (author).

The study sites were declared nature reserves in the 1940s and have been protected since as they contained the island’s best preserved native forest. Both sites experience a super-humid mesothermal climate (Halais & Davy, 1969) and have dense forest canopy that reaches 18 to 21 m (Vaughan & Wiehe, 1937; Lorence & Sussman, 1986). At each site, we compared two adjacent forest types: (i) a fenced Conservation Management Area (CMA) where woody IAP were removed in the 1990s (hereafter “weeded forest”), and which have since received maintenance weeding to preclude re-invasion (Fig. 1C; Florens & Baider, 2013), and (ii) the surrounding forest that has never been weeded and remains invaded (Figs. 1B, 1D). Because all best-preserved native wet forests in Mauritius are now heavily invaded by IAP (Florens et al., 2016), frugivory under pre-invasion conditions cannot be directly assessed. We therefore used a field experiment setting where IAP-invaded forests are compared with similar forests that have been weeded of IAP since the 1990s, to infer the influence of the presence of IAP on frugivory, following the approach of D’Antonio, Meyerson & Denslow (2001).

The CMA at Mare Longue covers approximately 3.46 ha, whereas the CMA at Brise Fer covers around 19 ha. To ensure comparability between sites, we selected a 2 ha section within the Brise Fer CMA where native vegetation is most comparable to that of Mare Longue CMA. Forest structure at both sites is broadly comparable between weeded and invaded patches in terms of native tree composition (Florens, 2008; Florens et al., 2012, 2017b). In 2007, Brise Fer invaded forest had 8,000 native stems ≥1 cm DBH per hectare, with 69 native species (Baider & Florens, 2011; Florens et al., 2012, 2017b), while the weeded forest had 6,920 stems per hectare with 69 native species (Florens, 2008). At Mare Longue, invaded forest contained 9,760 native stems ≥1 cm DBH per hectare from 67 native species, while the weeded forest had 8,480 stems per hectare from 62 native species (Florens, 2008; Florens et al., 2012, 2017b). Invaded forest supported extremely high densities of IAP, reaching 27,287 stems ≥1 cm DBH per hectare at Brise Fer and 52,920 stems per hectare at Mare Longue (Florens et al., 2017b). Strawberry Guava (*Psidium cattleyanum* Sabine) is the dominant invasive species, accounting for up to 95% of alien stems in lowland wet native forests of Mauritius (Florens et al., 2016). Other invasive species occur at far lower stem densities, including fleshy fruited species like *Ligustrum robustum* (1.5% of alien stems), *Litsea glutinosa* (0.8% of stems), *Wikstroemia indica* (0.2% of stems) or *Miconia leamarginata* (0.1% of stems).

### Frugivory rate

We adapted the protocol of the ‘global plasticine fruits experiment’ (Supplemental Material SM1) used to investigate plant–animal interactions using fruit models on islands (Galetti, Traveset & Jordano, 2021). Frugivory rates were quantified using artificial fruit models (~10 mm in diameter) made from red non-toxic modelling clay (Fig. 2), a technique widely used in ecological studies to assess avian frugivory (Alves-Costa & Lopes, 2001; Gagetti, Piratelli & Piña-Rodrigues, 2016; Ferger et al., 2016; Balasa et al., 2023). Red was chosen because it represents the most abundant group of fleshy fruit found throughout the world, is frequently associated with avian frugivory and is closer to those of naturally bird-dispersed fruits (Alves-Costa & Lopes, 2001; Duan, Goodale & Quan, 2014; Gagetti, Piratelli & Piña-Rodrigues, 2016; Hazell et al., 2023). In Mauritius, the selected colour and shape closely resemble the ripe fruits of endemic species such as *Ixora parviflora* (Rubiaceae) and *Syzygium glomeratum* (Myrtaceae) and also mimics small ripe fruits of the dominant IAP *P. cattleyanum*. Thus, the models used reflect ecologically relevant fruit traits present in both native and invaded forest conditions.

**Figure 2:**
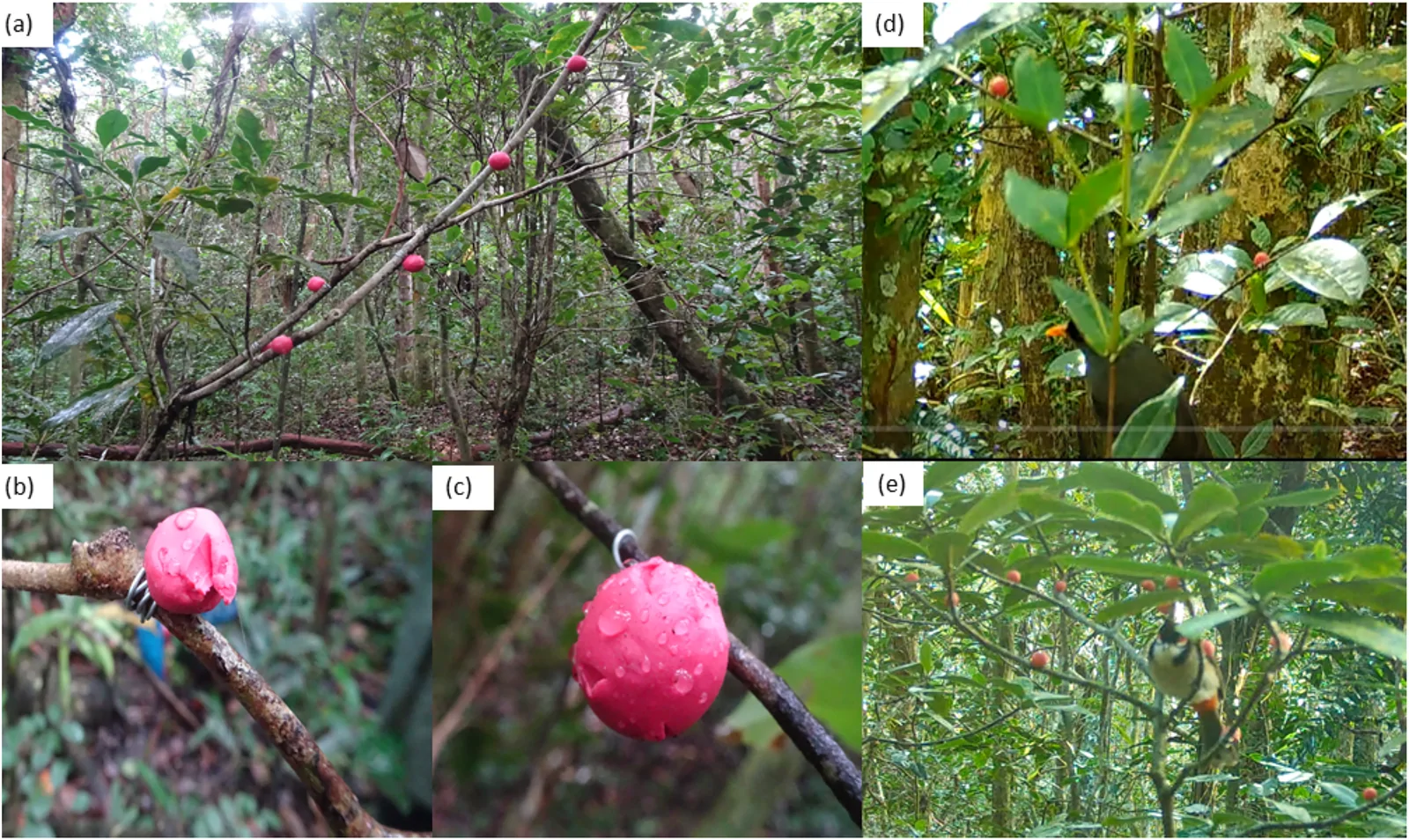
Clay fruit models used to assess avian frugivory. (A) Clay fruit models attached to branches with metal wire; (B and C) Clay models with beak marks; (D) Mauritius Bulbul (*Hypsipetes olivaceus*) attacking clay fruit model; (E) Red-whiskered Bulbul (*Pycnonotus jocosus*) attacking clay fruit model. Photo credit: Ghanishta Seeburrun.

Within each forest type, 12 understory native plants (1.5–2 m tall), irrespective of plant species identity, lacking natural fruits or flowers and spaced 10–30 m apart, were selected to ensure methodological consistency across forest types. On each plant, 15 fruit models were attached to branches at about one to two meters above the ground, with inter-fruit spacing of 5–30 cm. The objective was to provide standardised fruit units to quantify relative attack rates under comparable forest types as recommended by Galetti, Traveset & Jordano (2021). This resulted in a total of 180 fruit models per forest type at each study site. Within Brise Fer, the weeded and invaded forest plots were separated by approximately 250 m, while at Mare Longue the two plots were separated by approximately 300 m.

The fruit models were exposed for 72 h and then examined for peck or bite marks, indicating that a potential frugivore had attempted to consume them. The taxonomic identity of the frugivore that attempted to consume the fruit models was determined from the characteristic marks left on the models. Birds leave V-shaped beak marks, arthropods leave cut-shaped marks and dots, and mammals leave incisor-shaped or square-shaped tooth marks (Fig. S1). This experiment was conducted from August 2024 to July 2025, capturing seasonal variations in avian frugivory rate. In each sampling month, fruit models were placed in different randomly chosen plants in different areas of the forests to avoid habituating birds and to minimise exposure to the same birds through time, should they be territorial. Since our focus was on avian frugivory, we removed from the analysis fruit models with bite marks from mammals (all mammal teeth marks were from alien species, *N* = 16), arthropods (*N* = 116), unknown taxa, or those marks that were unidentifiable (*N* = 8), as well as models that were missing (*N* = 14). There was no attack by reptiles. To assess canopy openness, we took photographs 1 m above each understorey plant and analysed them using HabitApp on a smartphone (MacDonald, 2024).

### Frugivorous bird assemblages

Passive acoustic monitoring (PAM) was used to identify vocalising bird species in forests undergoing restoration and invaded forests simultaneously to the fruit model experiment. Acoustic data were collected using automatic recording units (ARUs) known as AudioMoth v1.2.0 recorders, which can record sound within a 50 to 100 m radius (Buchmann & Schurr, 2025). The recorders were set to record sound for 1 min every 5 min at a sampling rate of 48 kHz and medium gain (Hill et al., 2018). Two acoustic recorders were deployed per forest type to minimise effects of potential equipment failure at approximately 2 m above ground. We sub-sampled 1-min recordings corresponding to the morning bird chorus (from 30 min before sunrise to 4 h after sunrise) where the highest number of bird species vocalise (Catchpole & Slater, 2003; Metcalf et al., 2021). We restricted our analyses to functionally frugivorous bird species—those that rely primarily on fruits (obligate frugivores), include fruits as a substantial part of their diet (partial frugivores), or consume fruits opportunistically (opportunistic frugivores) (Kissling, Rahbek & Böhning-Gaese, 2007; Heinen et al., 2023). Each 1-min recording was visually examined through manual spectrogram scanning and listening to bird calls and songs using Kaleidoscope Pro 5.6.6 software (Wildlife Acoustics, 2024). When the species producing a vocalisation was uncertain, recordings were compared with reference calls and songs from the online sound database like Macaulay Library (Cornell Lab of Ornithology, https://www.macaulaylibrary.org). Recordings containing noise such as rain, strong wind and anthropogenic disturbances were excluded from further analysis.

For each frugivorous bird species, we calculated the acoustic ‘Detection Rate’ (DR) as the proportion of 1-min recordings in which the species was detected, which was used as a proxy for bird assemblages (Blake, 2021; Shaw et al., 2021; Hutschenreiter et al., 2024; Doohan et al., 2026). Acoustic Indices (AIs) were used to analyse how overall bird activity reflects intensity of frugivory (attack on clay models). This approach follows other recent studies where acoustic indices were used as a proxy to infer bird foraging attempts on artificial models (Alcocer et al., 2022; Schillé et al., 2024, 2025). For each 1-min recording, we calculated the following AIs: Acoustic Complexity Index (ACI) (Pieretti, Farina & Morri, 2011), Acoustic Diversity Index (ADI) (Villanueva-Rivera et al., 2011), Bioacoustic Index (BI) (Boelman et al., 2007), and the Normalised Difference Soundscape Index (NDSI) (Kasten et al., 2012). The indices were computed using Kaleidoscope Pro software, between a frequency range of 0–10 kHz, in which most bird species at our study locations vocalise. The spectral analysis of the signals was performed using a 1,024-point Fast Fourier transform (FFT). To calculate the ACI, the cluster size was adjusted to 10 s. ADI was computed using a 1,000 Hz frequency step and a −50 dB threshold, following the software’s default settings. For each forest type and month, we pooled all 1-min recordings from the three morning sampling days and calculated the median acoustic index value (Dröge et al., 2021; Schillé et al., 2025), yielding a single monthly measure of acoustic activity per forest type.

### Data analyses

#### Frugivory rate and frugivorous bird assemblages

The overall avian frugivory rate was calculated as the proportion of attacked artificial fruits relative to the total number of fruits deployed per forest type (180 fruits per month). Differences in overall frugivory rate between weeded and invaded forests were tested using paired Wilcoxon signed-rank tests based on monthly paired observations collected simultaneously within each site. Total bird and species-level differences in detection rates between weeded and invaded forests were tested using paired Wilcoxon signed-rank tests, with month treated as the paired unit as data were not normally distributed. To account for multiple testing across species, *p-*values were adjusted using a false discovery rate (FDR) correction. Frugivorous bird assemblages was analysed using non-metric multidimensional scaling (NMDS) based on monthly acoustic detection rates. These values were used to construct a community matrix, with rows representing site–monthly observations forest types and columns representing bird species, and detection rate as the response variable.

Community dissimilarity between forest types was quantified using a permutational multivariate analysis of variance (PERMANOVA) and adonis2 from the package ‘vegan’ in R (Oksanen et al., 2013) with 999 permutations and Bray–Curtis distances. Ordination quality was assessed using the stress value, with values <0.2. To ensure that any detected differences in frugivorous bird assemblages were not driven by unequal multivariate dispersion among forest types, homogeneity of dispersion was tested using betadisper followed by permutation tests. Species contributions to the ordination were examined using vector fitting (envfit), which fits species detection rates onto the NMDS configuration and identifies species whose detection patterns are significantly correlated with the ordination axes. Only species with significant vector fits (*p* < 0.05, based on 999 permutations) were retained for interpretation and visualisation. Spearman rank correlation analyses were conducted to examine associations between individual acoustic indices and total bird detection rate, both across all recordings and separately by forest type, to assess their relevance as indicators of avian activity.

#### Drivers of frugivory

Drivers of frugivory were analysed using generalised linear mixed models (GLMMs) fitted in the glmmTMB package (Brooks et al., 2017). Fruit attack rates were modelled using a beta-binomial error distribution with a logit link function. The response variable was specified as the number of attacked and intact fruits per tree. This approach models the probability of fruit attack while accounting for variation in the total number of fruits deployed per tree. To test the drivers of frugivory, the following predictors were included as fixed effects: forest type, (invaded and weeded), acoustic indices (ACI; ADI) and canopy cover (%). Correlations among acoustic indices were examined to identify redundancy. These exploratory analyses were used solely as a screening step to inform variable selection and avoid collinearity in subsequent models.

To account for non-independence arising from the sampling design, a random intercept was included for patch-month, defined as the unique combination of: Site × Forest type × Month. This patch structure accounts for trees sampled in each forest management site in a given month and sharing the same acoustic environment. Because trees were not repeatedly sampled across months (new trees were selected each month), tree identity was not included as a random effect. Model selection followed a backward elimination procedure, comparing nested models using likelihood ratio tests and Akaike’s Information Criterion (AIC). Predictors that did not improve model fit were removed sequentially. Pearson residuals and simulation-based residual diagnostics were conducted using the DHARMa package (Hartig, 2016). Marginal and conditional R^2^ values were calculated using the performance package to quantify variance explained by fixed effects alone and by the full mixed model, respectively. All statistical analyses were conducted in R version 4.3.2 (R Core Team, 2023).

### Ethics statement

AudioMoth recorders were deployed within study sites located in the National Park, in areas primarily used for research activities and invasive plant management. Although public access tracks exist within the park, they do not provide direct access to the study sites, resulting in minimal human presence. The devices were configured to record bird vocalisations only, thereby minimising the likelihood of capturing human speech. Each recorder was clearly labelled with contact details and the affiliated research institution to ensure transparency. Given the remoteness of the study sites and their limited accessibility, the risk of recording identifiable human data was considered negligible. As such, no additional ethical approval relating to human subjects was required. The study was approved by the National Parks and Conservation Service (NPCS), Ministry of Agro-Industry and Food Security, and conducted under permit reference NP Research/ProjV2.

## Results

### Attack rate on fruit models

Of the 7,920 fruit models deployed, 7,906 (99.8%) were retrieved. Overall, 535 fruit models (6.8%) showed evidence of bird pecking. Avian attempted attack rates were significantly higher in weeded forest than in invaded forests at both study sites, with BF showing higher rates (0.15 ± 0.02 *vs*. 0.04 ± 0.01; paired Wilcoxon signed-rank tests: V = 66, *p* < 0.0038) and ML showing a similar pattern (0.06 ± 0.01 *vs*. 0.02 ± 0.004; paired Wilcoxon signed-rank tests: V = 45, *p* = 0.0092) (Fig. 3; Table S1). Monthly frugivory rates were consistently higher in weeded forest than in invaded forest at both Brise Fer and Mare Longue (Fig. 4A). Moreover, throughout the study period, the proportional contribution of weeded forests to total monthly frugivory remained consistently high at both sites (Fig. 4B).

**Figure 3:**
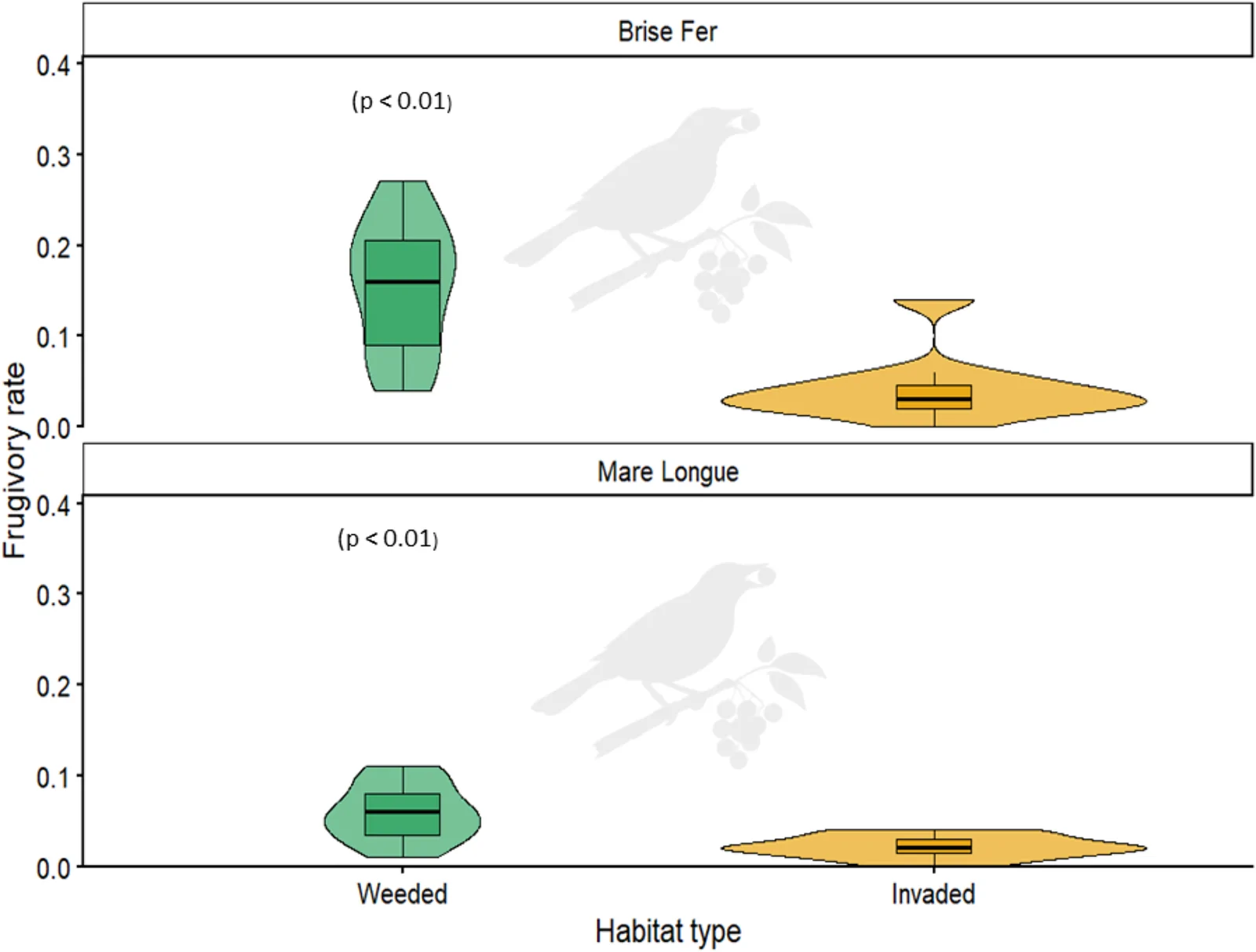
The distribution of attempted avian attack rates on fruit models in weeded and invaded forests at Brise Fer and Mare Longue, with embedded boxplots showing medians and interquartile ranges. Forest type is indicated by colour: green represents weeded forest and yellow represents invaded forest.

**Figure 4:**
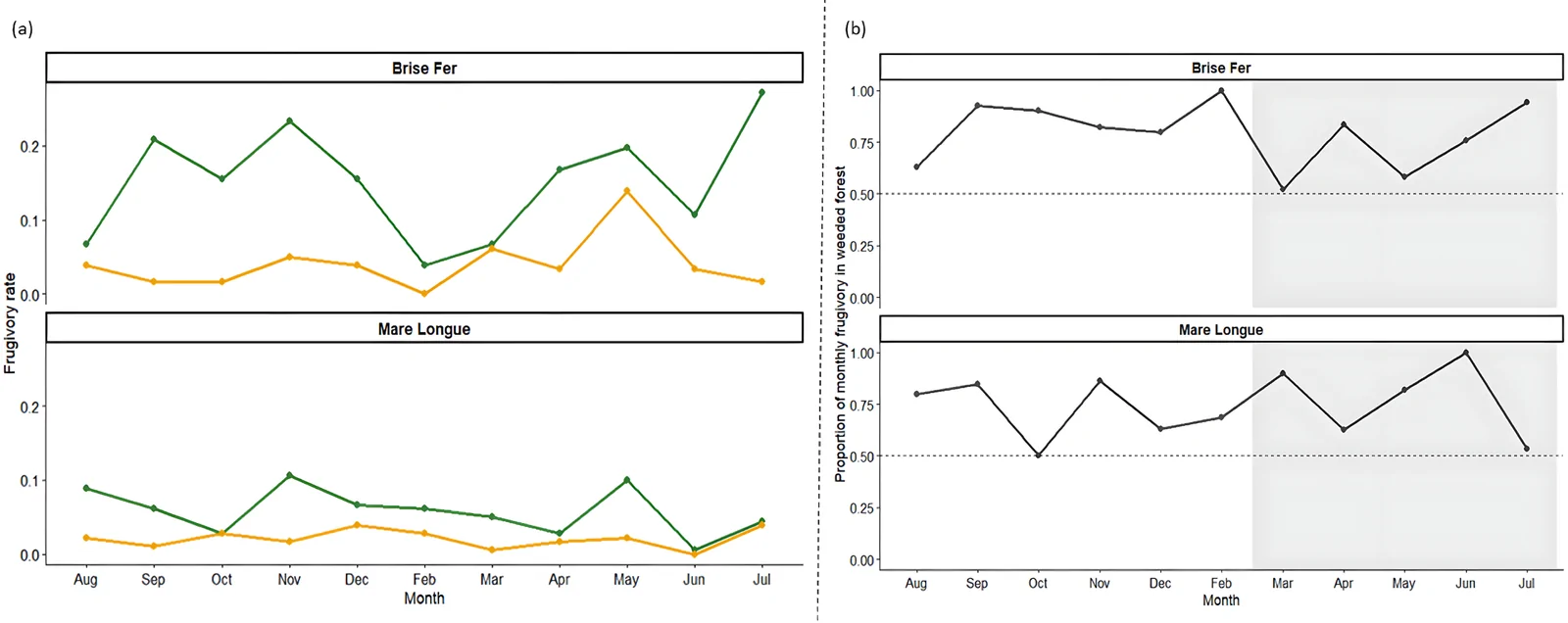
Seasonal patterns of avian frugivory in weeded and invaded forests. (A) Monthly variation in avian frugivory rates in weeded and invaded forests at Brise Fer and Mare Longue. Lines show monthly frugivory rates, calculated as the proportion of fruits consumed relative to the total number of fruits deployed, in weeded (green) and invaded (yellow) forests at each site; (B) proportional contribution of weeded forest to total monthly avian frugivory at Brise Fer and Mare Longue. Values represent the proportion of fruits attacked in weeded forest relative to the total number of fruits attacked in both weeded and invaded forests for each month. The dashed horizontal line indicates equal distribution of attacks in weeded and invaded forests (0.5). Values above 0.5 indicate more attacks in weeded forest for that month. Grey shading denotes the Strawberry Guava ripe fruit season (March–July).

### Bird detection patterns and frugivorous assemblages

Eighty-eight hours of recordings were obtained during the dawn chorus window, after excluding two months of missing data at each site due to recorder malfunction. After removing noise, we analysed 63.8 and 33 h of recordings from BF and ML, respectively. Twelve bird species were detected across both sites (Table S2), including nine frugivores, one carnivore, one insectivore and one granivore. These birds comprise the total number of known species in the studied site. Total detection rates of frugivorous birds were significantly higher in weeded forests than in invaded forests during every monthly survey at both sites throughout the survey period (paired Wilcoxon signed-rank tests; Brise Fer: V = 45, *p* = 0.004; Mare Longue: V = 45, *p* = 0.004) (Fig. 5). Frugivorous bird assemblages differed significantly between the two forest types at both BF (adonis2, Bray–Curtis; stress = 0.125; PERMANOVA: R^2^ = 0.27, F = 5.95, *p* = 0.002; Tables S3A and S3B) and ML (adonis2, Bray–Curtis; stress = 0.069; PERMANOVA: R^2^ = 0.18, F = 3.41, *p* = 0.007; Tables S3A and S3B). The substantial overlap observed in ordination space indicates partial assemblage turnover in invaded forests with shifts in species composition rather than complete species replacement (Fig. 6).

**Figure 5:**
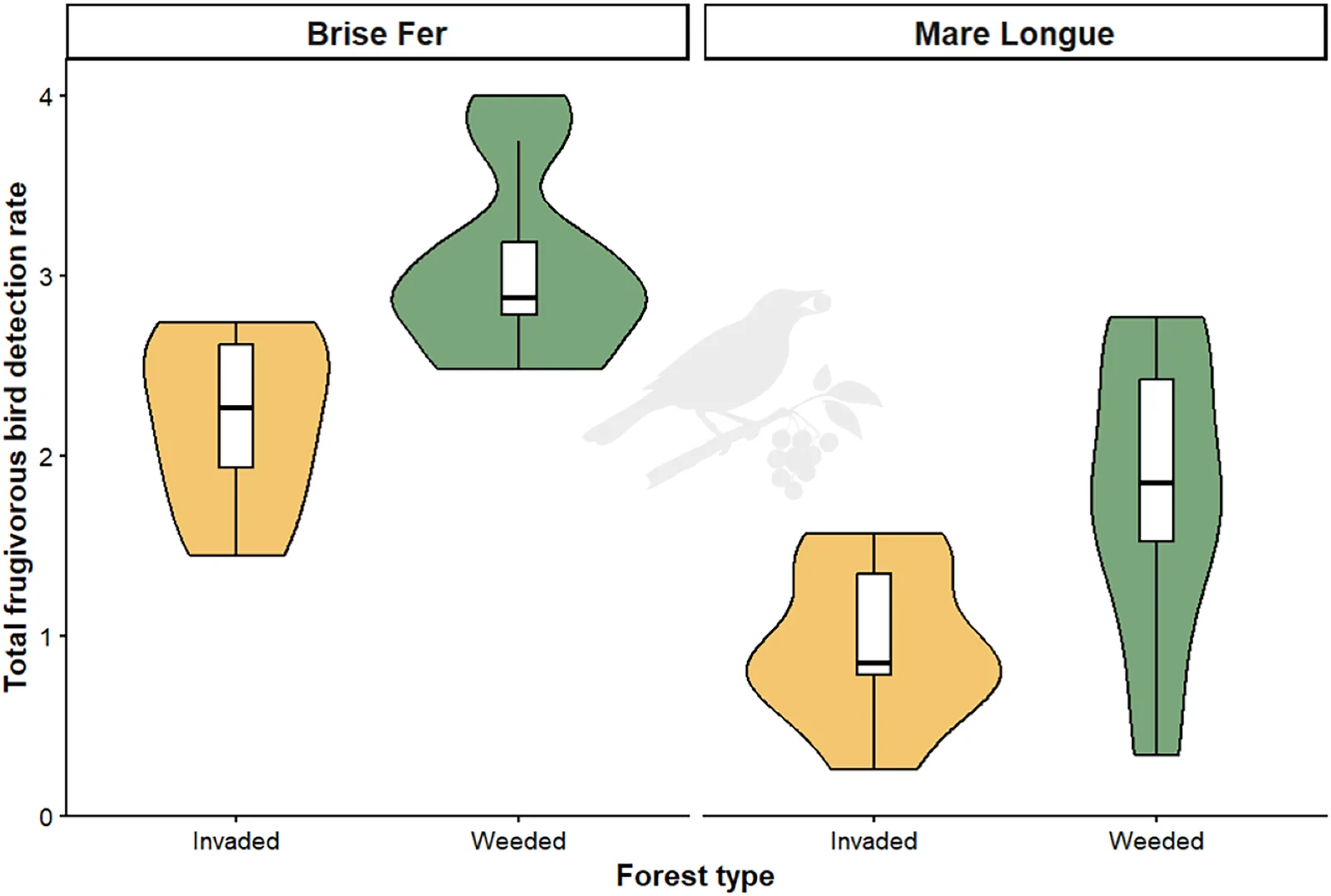
Distribution of total detection rates of frugivorous birds in invaded (orange) and weeded (green) forests at Brise Fer and Mare Longue. Detection rate was calculated as the total number of frugivorous bird detections divided by the total number of valid 1-min recordings.

**Figure 6:**
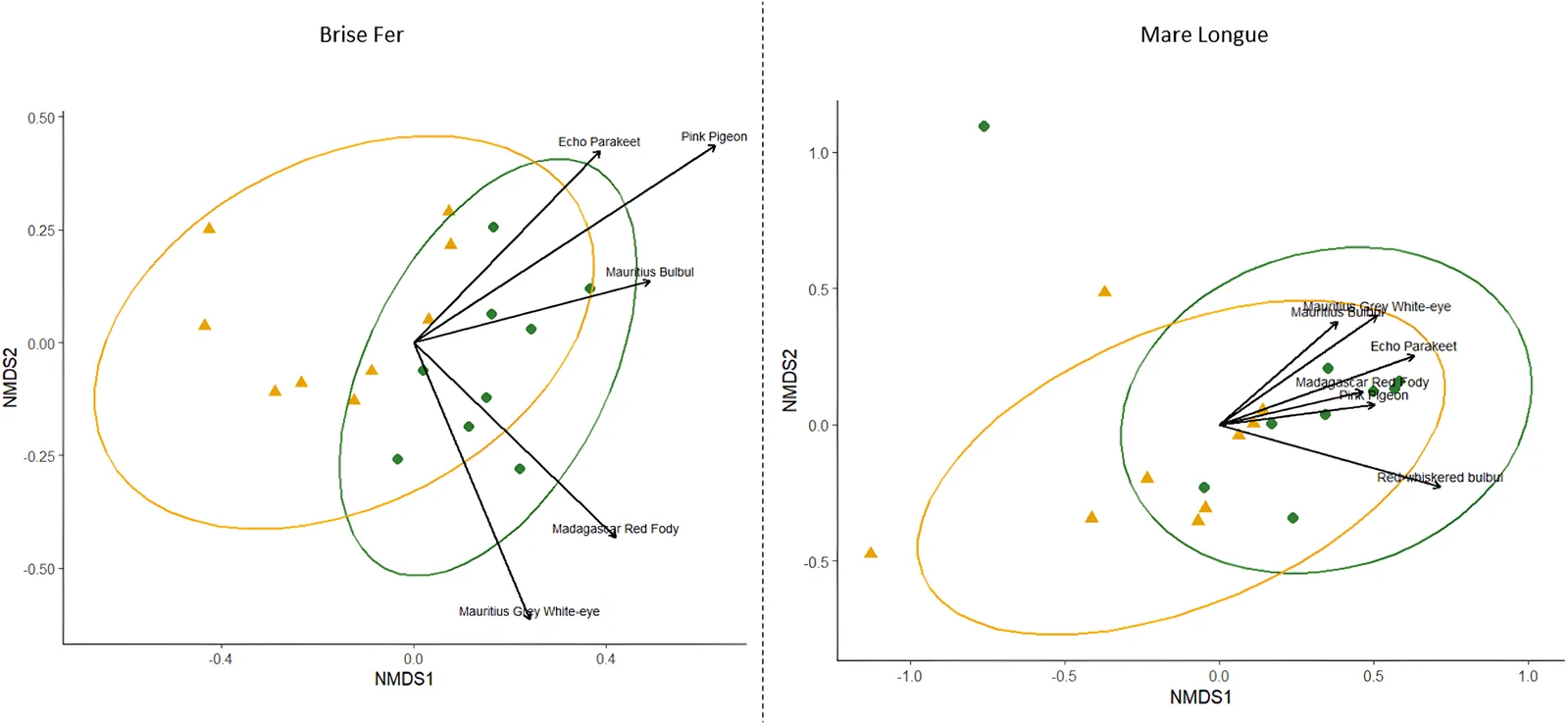
NMDS ordination plot of the compositions of bird communities at Brise Fer and Mare Longue for forest cleared of IAP (green) and invaded forest (yellow). Ellipses show the 95% confidence around forest types centroids. Arrows (envfit results, *p* < 0.05) represent species contributing strongly to the pattern that separates communities.

At BF, five frugivorous species and, at ML, six species contributed significantly to assemblage dissimilarity between forest types (Table S4). Moreover, detection rates of endemic birds were significantly higher in weeded forests than in invaded forests at both sites (paired Wilcoxon signed-rank tests; Brise Fer: *p* = 0.004; Mare Longue: *p* = 0.004), whereas detection rates of alien species did not differ significantly between forest types (Brise Fer: *p* = 0.055; Mare Longue: *p* = 0.426) (Fig. 7). The alien Red-whiskered Bulbul (*Pycnonotus jocosus*) was the most frequently detected species in both forest types, while the endemic Mauritius Bulbul (*Hypsipetes olivaceus*) exhibited significantly higher detection rates in weeded forests (Fig. S2; Table S5). Correlation analyses showed that only ADI and ACI were both positively correlated with total bird detection rate (ADI: ρ = 0.55, *p* < 0.001; ACI: ρ = 0.69, *p* < 0.001; Fig. S3; Table S6). Accordingly, ADI and ACI were retained as proxies of avian activity in subsequent generalised linear mixed model (GLMM) analyses.

**Figure 7:**
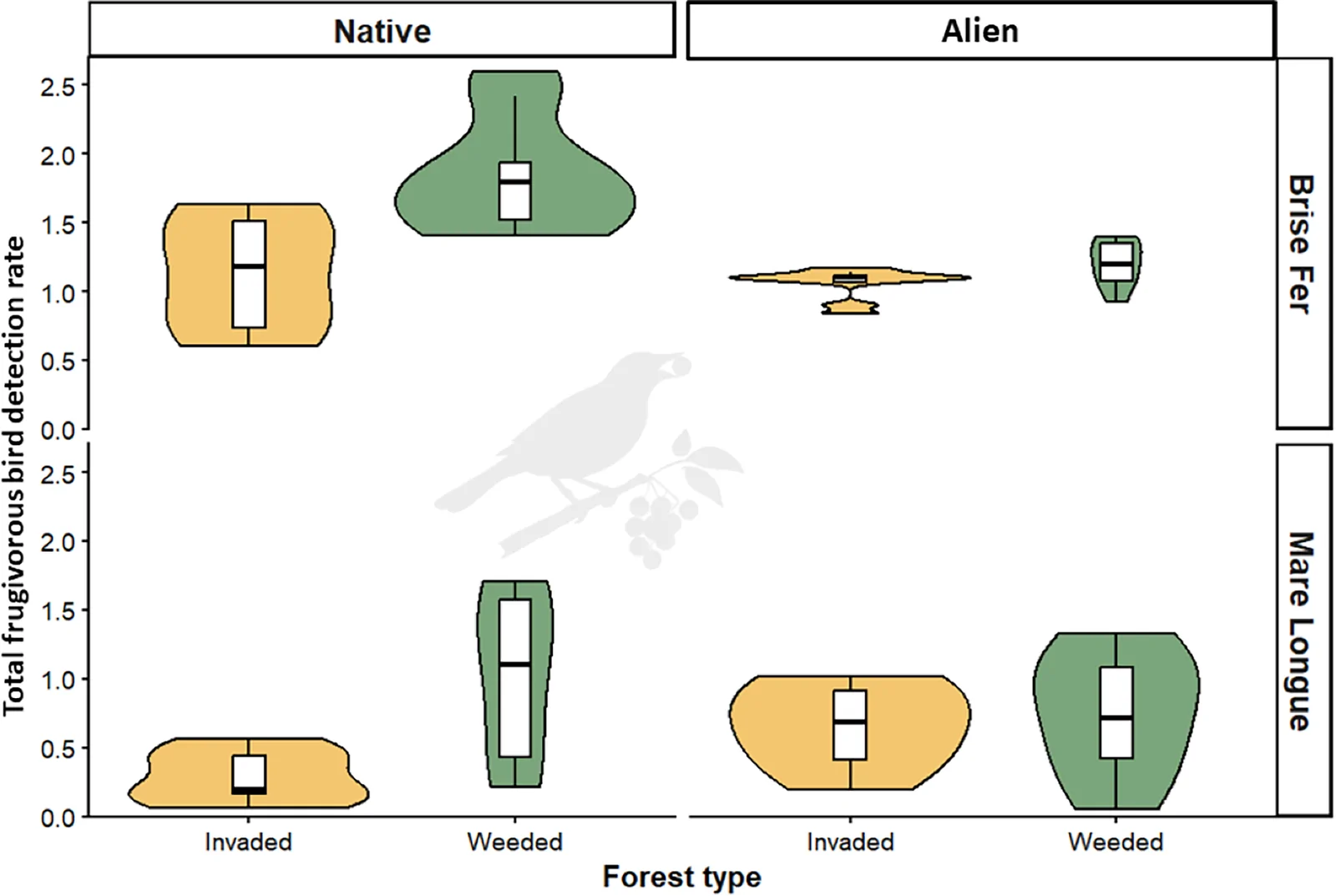
Distribution of pooled detection rates of native and alien frugivorous birds in weeded (green) and invaded forests (orange) at Brise Fer and Mare Longue. Detections were summed across all native or alien frugivorous bird species and divided by the total number of valid recording minutes.

### Drivers of frugivory rate/attack on fruit models

GLMM revealed strong effects of forest type and ACI on avian attack rates on fruit models. In the final beta-binomial GLMM, fruit models placed in weeded forests had significantly higher attack probabilities than those in invaded forests (β = 1.22 ± 0.20 SE, z = 6.09, *p* < 0.001). This corresponded to approximately a threefold increase in the odds of fruit attack in weeded forests compared to invaded forests (odds ratio = 3.38, 95% CI [2.28–5.01]) (Fig. 8; Table S7). The acoustic indices ACI and ADI were highly correlated (Pearson r = 0.90, *p* < 0.001) and were therefore analysed in separate models to avoid multicollinearity. Both indices were positively associated with fruit attack probability, but the model including ACI provided a better fit to the data and was retained as the acoustic predictor in the final model.

**Figure 8:**
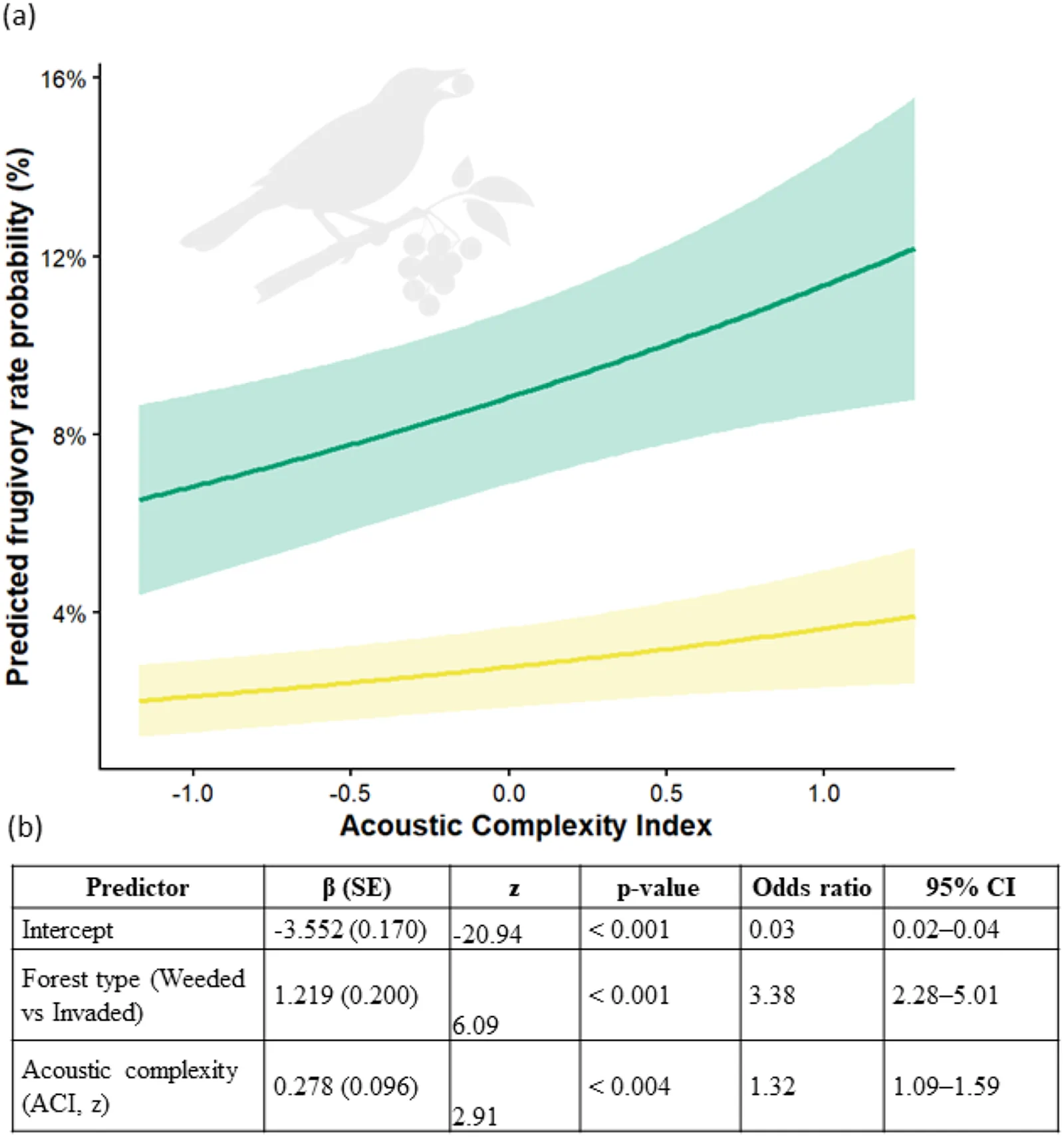
Drivers of avian frugivory in forest cleared of invasive alien plants (IAP) and invaded forest. (A) Model-predicted frugivory rate in relation to acoustic complexity (ACI) based on the beta binomial GLMM. Lines represent predicted values and shaded bands indicate 95% confidence intervals. Green colour represent forest cleared of IAP and yellow colour represent invaded forest. Predictions are expressed as attacked fruits; (B) Fixed-effect estimates from the final GLMM, showing regression coefficients (β), standard errors (SE), odd ratios, and 95% confidence intervals.

In the final model including ACI, fruit attack probability increased significantly with increasing acoustic complexity (β = 0.28 ± 0.10 SE, z = 2.91, *p* = 0.004). A one-standard-deviation increase in ACI was associated with approximately 32% higher odds of fruit attack (odds ratio = 1.32, 95% CI [1.09–1.59]; Fig. 8; Table S7). Canopy cover did not significantly influence fruit attack probability (β = 0.02 ± 0.08 SE, z = 0.24, *p* = 0.81) and did not improve model fit during model selection (likelihood ratio test *p* = 0.81; ΔAIC = +1.94), and was therefore excluded from the final model. Model diagnostics indicated good model performance. Simulation-based residual diagnostics showed no evidence of overdispersion (dispersion = 1.02, *p* = 0.84), no zero inflation (*p* = 0.74), and no significant outliers (Fig. S4). The model explained a moderate proportion of the variance in fruit attack probability, with marginal R^2^ = 0.29 and conditional R^2^ = 0.36.

### Discussion

Overall, our findings are consistent with the hypotheses that invasion by woody IAP is associated with lower frugivorous activity and reduced frugivory in the lowland wet forests studied. Frugivorous bird community composition differed between forest types, with endemic frugivores being significantly more common in weeded forests than in invaded forests. Consistent with these patterns, attack rates on artificial fruits were approximately three times higher in weeded forests, indicating stronger avian frugivory where IAP had been removed. Furthermore, the ACI was positively associated with fruit attack rates, suggesting that acoustic monitoring may provide a useful proxy for frugivore activity. Together, these results indicate that invasion by understory IAP (chiefly *P. cattleyanum*) in the dominant vegetation type of Mauritius (lowland wet forests) would reduce habitat quality for native frugivores and weaken mutualistic interaction like frugivory.

#### Biological implications

Invasion of native forests mainly by understory Strawberry Guava results in lower abundances of frugivorous birds and modification of their assemblages, especially for native birds. This pattern aligns with previous studies showing that IAP often restructure bird community composition (Hejda et al., 2017; Mangachena & Geerts, 2017; Grzędzicka & Reif, 2020; Da Silva et al., 2020). This indicates that invasion may reduce habitat quality for most birds. Strawberry Guava at the study sites reaches extremely high densities (Florens et al., 2016), a situation known to reduce forest penetrability in the understorey by some birds (Osborne, Sparling & Hopkins, 2012). Furthermore, invasive plants also reduce fruit production among native fleshy fruited species (Baider & Florens, 2006; Monty, Florens & Baider, 2013; Krivek et al., 2020) and birds are known to typically concentrate their foraging and overall activity where food is most accessible, abundant, and energetically profitable (Traveset & Richardson, 2006; Smith & McWilliams, 2014; Stewart et al., 2021). Additionally, alien-invaded forests host fewer species and individuals of native butterflies (an often used indicator group for insect diversity) compared to nearby weeded forests (Florens et al., 2010) which may further reduce habitat quality for the birds, because many of them also eat insects (Table S3), particularly during the breeding season (September to March) when protein-rich preys are essential for chick development (Cheke, 1987; Bissessur, Probst & Florens, 2017). Therefore, alien plant invasion in native habitats could reduce habitat quality for native frugivorous birds, despite the principal alien plant (Strawberry Guava) being a fleshy-fruited species itself.

Birds attacked fruit models three times less frequently in invaded forests compared to adjacent weeded forests. Fruit models are designed to mimic natural fruits to quantify frugivory rate. Therefore, lower attack rates indicate reduced bird–fruit interactions and, by extension, lower avian frugivory in invaded habitats. For mutualistic interactions to occur, species must meet within their habitat, itself a process largely determined by species abundance as well as the habitat use and behaviour of the interacting taxa (McConkey & O’Farrill, 2015; Heinen, Rahbek & Borregaard, 2020). We showed that lower fruit attack rates were associated with lower ACI values in invaded forests compared to weeded forests. As ACI reflects avian vocal activity, lower values may indicate lower bird presence, which during the dawn chorus is often linked to birds’ intensified behavioural engagement while searching for potential food resources (Clay, Smith & Blumstein, 2012; Ramesh et al., 2025). In invaded forests, where native fruit resources are rarer (Monty, Florens & Baider, 2013; Krivek et al., 2020; Bissessur et al., 2023) and bird detection rates are lower, it is expected that birds will encounter fruits less frequently, which we confirmed through detecting reduced attack rates on fruit models. These findings highlight how IAP could alter bird activity, presumably as a result of altered habitat structure (*e.g*., a much higher stem density) that impedes flight and/or resource availability (fewer native fruits and insects), ultimately weakening plant–animal interactions (Traveset & Richardson, 2006; Smith & McWilliams, 2014; Stewart et al., 2021).

Seed dispersal starts with the pre-dispersal phase which involves frugivores visiting fruiting trees and consuming fruits (here reflected by attacks on fruit models) (Jordano, 2000; Wang & Smith, 2002; Jordano et al., 2007; Wehncke, 2010). Weakening of this first phase is expected to carry over through subsequent dispersal phases (Wehncke, 2010), thereby weakening the entire seed dispersal process, hence the regeneration of native plants (Jordano, 2000; Schupp, Jordano & Gómez, 2010). The endemic Mauritius Bulbul, in particular, was the species that was most negatively impacted by IAP. This bird forages on at least 17 native fleshy-fruited species and is the best pollinator of the threatened endemic *Roussea simplex*, one of the fastest declining species worldwide (Bissessur, Probst & Florens, 2017; Bissessur et al., 2019). Consequently, alien plant invasion could be inferred to weaken the bird’s important ecological role of aiding native plant reproduction. Some studies have shown that birds may forage more in invaded habitats when IAP are fruiting (Mokotjomela, Musil & Esler, 2013; Marciniak et al., 2024) and that this in turn potentially eases the pre-dispersal of co-occurring native species (Vergara-Tabares et al., 2021). However, such a trend did not show up in our study, where attack rates on fruit models were in all months lower in the invaded areas compared to the weeded areas, including during the peak fruiting period of the dominant invasive Strawberry Guava (March–July) (Fig. 4B). This indicates that even when fruiting, Strawberry Guava may not compensate for the weakening of native seed-dispersal services caused by IAP invasion.

#### Management implications

Bird conservation in Mauritius has primarily focused on species-centred interventions like captive breeding, supplemental feeding, reintroduction, and reinforcement (Simberloff, 1998; Jones, 2004; Edmunds et al., 2008; Cristinacce et al., 2009; Florens, 2013). These strategies are essential when species are at high risk of extinction (Black, 1991). However, despite intensive species-centred conservation since two to five decades depending on species, all originally threatened Mauritius endemic bird species remain threatened today (BirdLife International, 2026), stressing the need to deepen and/or broaden conservation strategies. We show that invasion by alien plants degrades the habitat quality for native frugivorous birds, reducing their carrying capacity compared to where IAP were weeded. The so far partially successful bird conservation projects must therefore shift a larger component of their efforts to controlling IAP over larger areas than the current ~700 ha (8.5% of remnant native habitats) (Republic of Mauritius, 2024), while also proceeding carefully whenever weeding may comprise downsides (Carpouron et al., 2023). This is particularly urgent because alien plant invasions are inexorably progressing (Florens et al., 2016) and because this worsening invasion is occurring over an area of native habitats that is over tenfold the area over which IAP have been controlled (Republic of Mauritius, 2024), resulting in an overall net loss of native biodiversity. Controlling IAP over larger areas is doubly judicious as it improves prospects for many other native species like lianas and epiphytes (Baguette, Baider & Florens, 2025b), native plants (Baider & Florens, 2011, 2022) or molluscs (Florens & Baider, 2007).

Beyond improving habitat quality for endemic birds, we show that habitat restoration after weeding IAP could strengthen native seed-dispersal mutualisms, echoing similar findings with the frugivorous bat *Pteropus niger* (Krivek et al., 2020; Seegobin, Oleksy & Florens, 2024, 2026). Expanding areas weeded of IAP would indeed contribute towards stemming and eventually reversing the current decline of fleshy-fruited species, whether threatened (Tatayah et al., 2022, 2025) or not (Baider et al., 2025) thereby strengthening the island’s remaining plant–frugivore networks (Heinen et al., 2023). This situation is also likely to help mitigate the recently worsening human-wildlife conflict generated by *P. niger* including commercial fruits in its diet (Florens, 2016; Florens & Baider, 2019). Indeed, as bat’s natural foraging habitat continues to degrade under the impacts of alien plants (Krivek et al., 2020) and animals (Seegobin, Oleksy & Florens, 2026), the bat has increasingly been relying on commercial fruits (Oleksy et al., 2021; Bhanda, Oleksy & Florens, 2026). Together, birds and bats play complementary roles in dispersing seeds of the majority of native fleshy-fruited plants (Florens et al., 2017a; Heinen, Rahbek & Borregaard, 2020). Reinstating and reinforcing seed dispersal mutualisms would also enhance natural regeneration which would further reduce the current costly and excessive reliance on nursery-based plant propagation in restoration programmes (Caves et al., 2013; Costa et al., 2022a; Baguette et al., 2026, unpublished data). Furthermore, our study underscores the crucial consideration of IAP control on projects seeking to reinstate lost plant–frugivore interactions, an often overlooked aspect in such projects (Derham et al., 2018).

Mauritius retains one of the most threatened biota worldwide (Botanic Gardens Conservation International, 2021), despite decades of intense conservation. Implementing more cost-effective conservation projects should be the priority. Improved monitoring would support this objective (FMMP Baguette et al., 2026, unpublished data), as would stopping the common practice of cutting native fleshy-fruited pioneer species from conservation areas (Baguette, Baider & Florens, 2025b). Our study further supports the validity of shifting conservation towards a less highly interventionist and species-centred approach (Florens, 2013; Seeburrun et al., 2026) to one fostering natural functioning and regeneration which is cheaper and better for native biodiversity (Baguette, Baider & Florens, 2025a). Following control of IAP, native plants often naturally regenerate fast, with native pioneer trees like *Harungana madagascariensis* (Florens & Baider, 2013). These rapidly mature and provide floral, fruit and other resources to fauna and flora (Heinen et al., 2023; Baguette, Baider & Florens, 2025a). However, reinvasion from surrounding invaded areas remains a risk (Singh & Byun, 2023), particularly when weeded areas are small hence even with their core remaining close to invaded habitats and sources of IAP propagules. Expanding weeded areas could both reduce reinvasion rates and shifting frugivore–plant interactions away from alien fruits toward native ones (Singh & Byun, 2023; Marciniak et al., 2024). Weeding larger areas could indeed increasingly shift the most frequently detected frugivore in both invaded and weeded forest (the alien Red-whiskered Bulbul) from being a problematic disperser of IAP (Linnebjerg et al., 2010) to fostering native forest regeneration (García et al., 2014; Seeburrun et al., 2026).

### Limitation of the study

PAM has been used as a non-invasive tool to quantify bird activity and assemblages (Pérez-Granados & Traba, 2021). It has been applied in recent studies as a proxy to investigate avian predation on artificial prey models (Schillé et al., 2024, 2025), and also provides essential information about plant–animal interactions (Dong et al., 2023). We used acoustic indices as proxies of bird activity to examine their relationship with frugivory. However, acoustic monitoring captures vocal activity rather than direct feeding behaviour. The species responsible for attacks on artificial fruits remains unknown. Acoustic indices should therefore be interpreted as indicators of overall bird activity that may influence encounter rates with fruit resources rather than direct measures of frugivory. Moreover, the low spatial replication (two study sites) is unfortunate but reflects the rarity of suitable weeded and invaded forest patches in Mauritius. Brise Fer and Mare Longue represent the only sufficiently large CMA suitable for this study on the island’s dominant native ecosystem (the lowland wet forest). This is because they are the only two sites that hold CMAs managed since the 1990s within this ecosystem which are large enough to minimise edge effect between the two contrasting forest types. Other weeded native habitat areas either belong to distinct vegetation types (*e.g*., mossy forest, heath, marshland, or stunted thicket) that represent exceptional native habitat types of the island (Vaughan & Wiehe, 1937) or were too small (*e.g*., 0.5 ha weeded area in Macchabé forest) to be useful.

Furthermore, the experiment was repeated over an 11-month period spanning both summer and winter seasons. The temporal observations do not replace independent spatial replication of weeded and invaded forest patches, but rather, the repeated sampling was used to assess whether differences between forest types were consistent through time and across seasons. Consequently, our findings should be interpreted as evidence from Mauritius lowland wet forests that are overwhelmingly dominated by Strawberry Guava invasion rather than as a comprehensive assessment of all invaded native vegetation communities in Mauritius, which also comprise mossy forest, heath, stunted thickets, marshy areas or dry coastal forest with often starkly different characters and species composition, including of invasive alien species. Nevertheless, the consistent pattern observed across both sites and throughout the sampling period lends confidence to the results despite limited site replication.

## Conclusion

We combined avian audio recordings with estimates of avian frugivory using attack rates on fruit models to compare native forests that are invaded and managed for IAP removal on the oceanic island of Mauritius. We found that despite the main IAP being the fleshy-fruited Strawberry Guava, alien plant invasion led to lower ACI of birds and a three-fold lower rate of attack on fruit models. We thus provide empirical evidence that understorey fleshy-fruited IAP may degrade the habitat of frugivorous birds and weaken the seed-dispersal mutualism involving birds.

In context, several implications for conservation management emerge. First, the study shows that even when invasive alien species bears many fleshy-fruits, its weeding does not equate to a degradation of the foraging habitat quality for frugivorous birds. Second, habitat restoration through IAP control should be given greater consideration in the endemic bird conservation programmes Mauritius is well-known for, as all seven bird species threatened in the 1990s are still threatened today, and our study indicates that most of them would directly benefit from IAP control. Third, IAP-control could reinforce mutualisms between native birds and plants which in turn would logically render the current expensive intensive conservation management even less necessary, thus freeing resources to increase native habitat areas where IAP are controlled. Fourth, the findings illustrate the importance of incorporating IAP control ahead of programmes of re-introduction of locally extinct frugivores or introduction of analogue species meant to reinstate lost ecological function like seed dissemination. Finally, the advanced state of IAP invasion on Mauritius can serve as a harbinger of what awaits other islands as invasion by alien plants progresses to approach the situation already prevailing on Mauritius.

## Supplemental Information

10.7717/peerj.21749/supp-1 Supplemental Information 1 Clay fruit models showing characteristic attack marks by different taxa. Fruit models numbered 1–27 display peck marks consistent with avian frugivory; models 28–30 show bite marks attributed to alien monkeys; models 31–34 show bite marks attributed to alien rats; and models 35–36 display damage consistent with insect attacks. Photo credit: Ghanishta Seeburrun.

10.7717/peerj.21749/supp-2 Supplemental Information 2 Mean detection rate (± SE) of frugivorous bird species in weeded (green) and invaded (yellow) forest plots at Brise Fer and Mare Longue. Species are ordered by decreasing detection rate in the weeded forest. Asterisks* indicate non-native bird species.

10.7717/peerj.21749/supp-3 Supplemental Information 3 Correlation between acoustic indices and total bird detection rate. (a) Correlation between acoustic diversity index (ADI) and total bird detection rate, (b) correlation between acoustic complexity index (ACI) and total bird detection rate

10.7717/peerj.21749/supp-4 Supplemental Information 4 DHARMa residual diagnostics for the generalised linear mixed model of frugivory. Quantile–quantile (QQ) plot of simulated residuals and residuals plotted against fitted values. Diagnostics indicate no significant deviations from model assumptions, with no evidence of overdispersion, outliers, or systematic patterns in residuals.

10.7717/peerj.21749/supp-5 Supplemental Information 5 Frugivory rates in weeded and invaded forests at Brise Fer and Mare Longue. (a) Mean monthly frugivory rates (± SE) calculated as the proportion of attacked fruits per tree, based on 11 months of sampling. (b) Paired Wilcoxon signed-rank tests comparing frugivory rates between weeded and invaded forests for each site. V denotes the Paired Wilcoxon signed-rank test statistic.

10.7717/peerj.21749/supp-6 Supplemental Information 6 Origin and diet composition of bird species recorded in the study.

10.7717/peerj.21749/supp-7 Supplemental Information 7 Effects of forest type on bird community composition at Brise Fer and Mare Longue. (a) Permutational multivariate analysis of variance (PERMANOVA) testing for differences in bird community composition between weeded and invaded forests based on Bray–Curtis dissimilarities (999 permutations). R^2^ indicates the proportion of variance explained by forest type. (b) Permutation tests for homogeneity of multivariate dispersions (PERMDISP), showing no evidence that PERMANOVA results were driven by differences in within-group dispersion.

10.7717/peerj.21749/supp-8 Supplemental Information 8 Species most strongly associated with community differences, based on permutation-based vector fitting (envfit). r^2^ values indicate the strength of association between species and community structure. Asterisks* represent non-native bird species. r^2^ values indicate the strength of association between species and community structure. Asterisks* represent non-native bird species.

10.7717/peerj.21749/supp-9 Supplemental Information 9 Mean monthly detection rates of bird species across weeded and invaded forests at Brise Fer and Mare Longue. Values represent mean detection rate ± standard error (SE), calculated as the proportion of 1-min recordings in which a species was detected. P-values were adjusted for multiple testing using the false discovery rate (FDR) method. Significant differences are in bold. Species marked with an asterisk (*) are non-native bird species.

10.7717/peerj.21749/supp-10 Supplemental Information 10 Acoustic indices in relation to bird detection rate. (a) Spearman rank correlations between acoustic indices and total bird detection rate. Spearman’s rank correlation coefficients (ρ) and associated p-values are shown for associations between acoustic indices (ACI, ADI, BI, NDSI) and total bird detection rate across all recordings; (b) Spearman rank correlations between acoustic indices and total bird detection rate by forest type. Spearman’s rank correlation coefficients (ρ) are shown for associations between acoustic indices and total bird detection rate calculated separately for weeded and invaded forests.

10.7717/peerj.21749/supp-11 Supplemental Information 11 Results of the final generalised linear mixed model (GLMM) examining drivers of frugivory.

10.7717/peerj.21749/supp-12 Supplemental Information 12 Artificial fruit experiment protocol.

## Additional Information and Declarations

### Data Availability

The following information was supplied regarding data availability:

The data is available at Zenodo: Seeburrun, G. (2026). Data [Dataset]. Zenodo. https://doi.org/10.5281/zenodo.18228318.

The code is available at Zenodo: Seeburrun, G. (2026). R code for data analyses and visualisation_Frugivory. Zenodo. https://doi.org/10.5281/zenodo.19060548.
