# Supplementary material for: Invasive Strawberry Guava weakens avian frugivory in native forests of an oceanic island
Source: PeerJ. 2026 Sep 30;14:e21749. doi: 10.7717/peerj.21749 (PMC13633687; doi:10.7717/peerj.21749)
Supplement: Supplementary file 2 [file peerj-14-21749-s004.pdf]

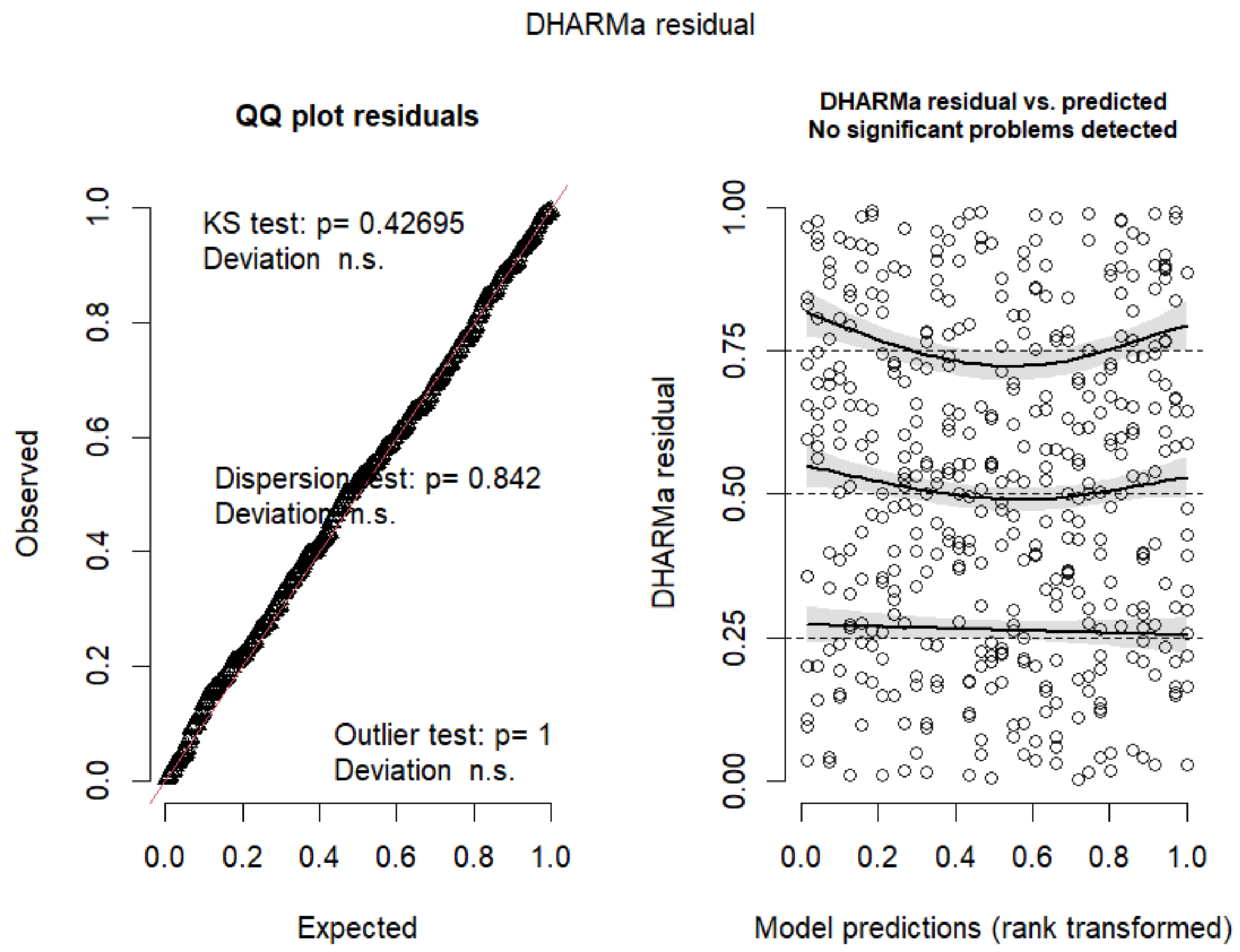
DHARMa residual
QQ plot residuals
KS test: p= 0.42695
Deviation n.s.
Dispersion test: p= 0.842
Deviation n.s.
Outlier test: p= 1
Deviation n.s.
Observed
Expected
0.0
0.2
0.4
0.6
0.8
1.0
DHARMa residual vs. predicted
No significant problems detected
DHARMa residual
0.00
0.25
0.50
0.75
1.00
Model predictions (rank transformed)
