# Supplementary material for: Invasive Strawberry Guava weakens avian frugivory in native forests of an oceanic island
Source: PeerJ. 2026 Sep 30;14:e21749. doi: 10.7717/peerj.21749 (PMC13633687; doi:10.7717/peerj.21749)
Supplement: Supplementary file 3 [file peerj-14-21749-s012.pdf]

Artificial Fruit Experiment Protocol

1. Study design

We adapted the protocol from the 'global plasticine fruits experiment' by Galetti, Traveset & Jordano (2021), which was used to investigate plant–animal interactions using fruit models on islands. Frugivory rates were quantified using artificial fruit models), a technique widely used in ecological studies to assess avian frugivory (Alves-Costa & Lopes, 2001; Gagetti, Piratelli & Piña-Rodrigues, 2016; Ferger et al., 2016; Balasa et al., 2023).

Artificial fruit experiments were conducted in the Black River Gorges National Park, Mauritius, at two study sites (Brise Fer and Mare Longue). Each site had two adjacent forest types: one forest invaded by invasive alien plants (IAP) (invaded forest) and one undergoing IAP removal (weeded forest) (Figure 1). Experiments were conducted monthly between August 2024 and July 2025.

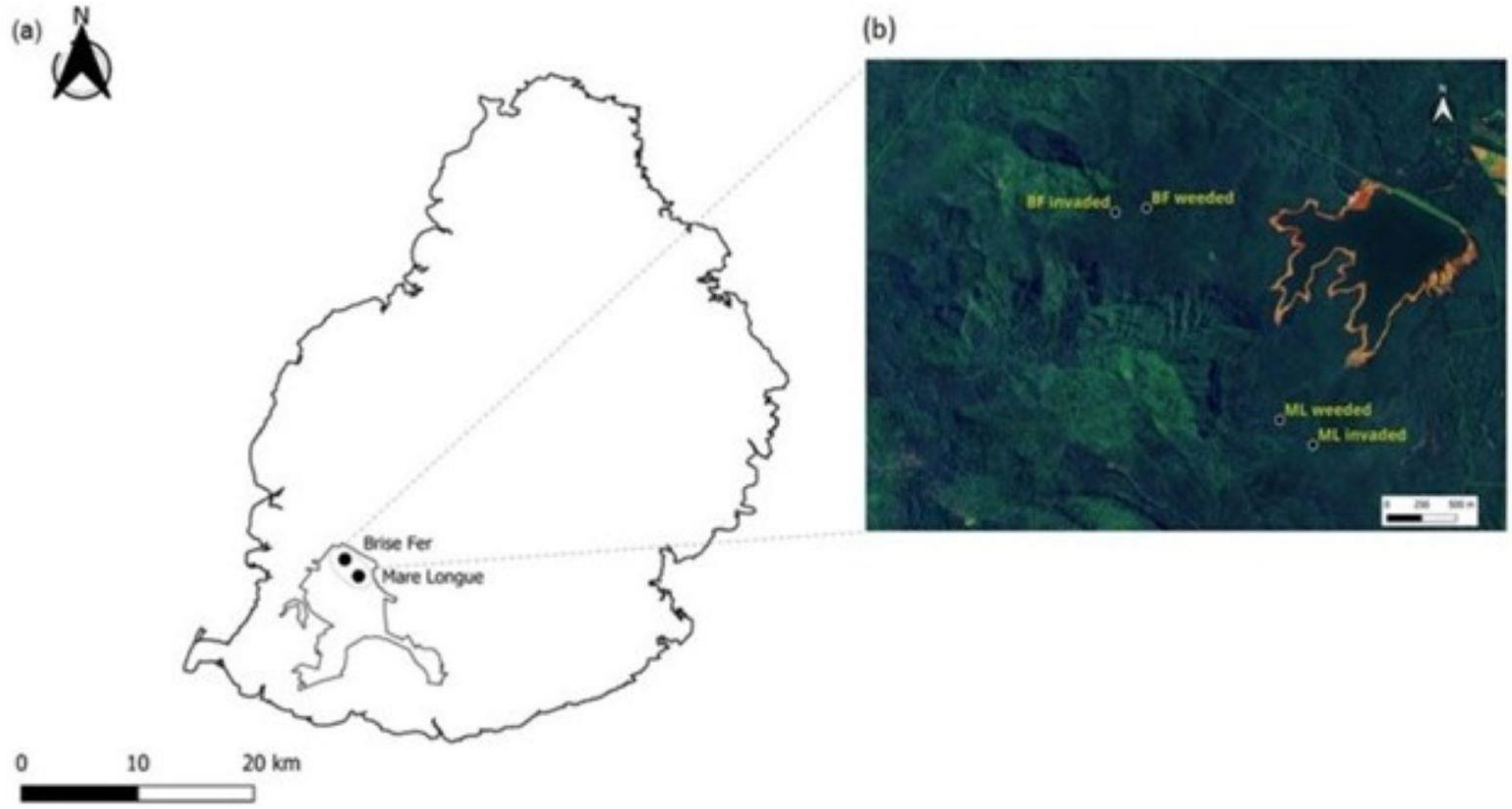

Figure 1. Location of the study sites within the Black River Gorges National Park (BRGNP), Mauritius. (a) Map of Mauritius showing the position of BRGNP and the study sites of Brise Fer (BF) and Mare Longue (ML). The Mauritius outline was digitised by the authors from © 2026 Google Earth Pro imagery; (b) Zoomed view of the study area indicating a center point of invaded and weeded forest plots. Plot locations were recorded using GPS during field surveys and mapped in QGIS version 3.34 using background satellite imagery © 2026 Google Earth Pro ; (c) Forest where alien plants have been cleared; (d) Forest invaded by alien plants, the reddish brown stems are that of the invasive Strawberry Guava (*Psidium cattleyanum*). Photo credit: Ghanishta Seeburrun (author).

2. Construction of artificial fruit models

Artificial fruits were constructed from odourless, non-toxic red modelling clay moulded into spherical fruits approximately 10 mm in diameter (Figure 2). The selected colour and shape

closely resembles the ripe fruits of endemic species such as *Ixora parviflora* (Rubiaceae) and *Syzygium glomeratum* (Myrtaceae) and also mimics small ripe fruits of the dominant IAP *P. cattleyanum*. Thus, the models used reflect ecologically relevant fruit traits present in both native and invaded forest conditions. Each fruit model was attached individually to a thin, flexible wire, allowing it to be securely attached to branches while permitting birds to peck the fruit naturally. Fingerprints or surface irregularities were smoothed before deployment to avoid introducing visual cues.

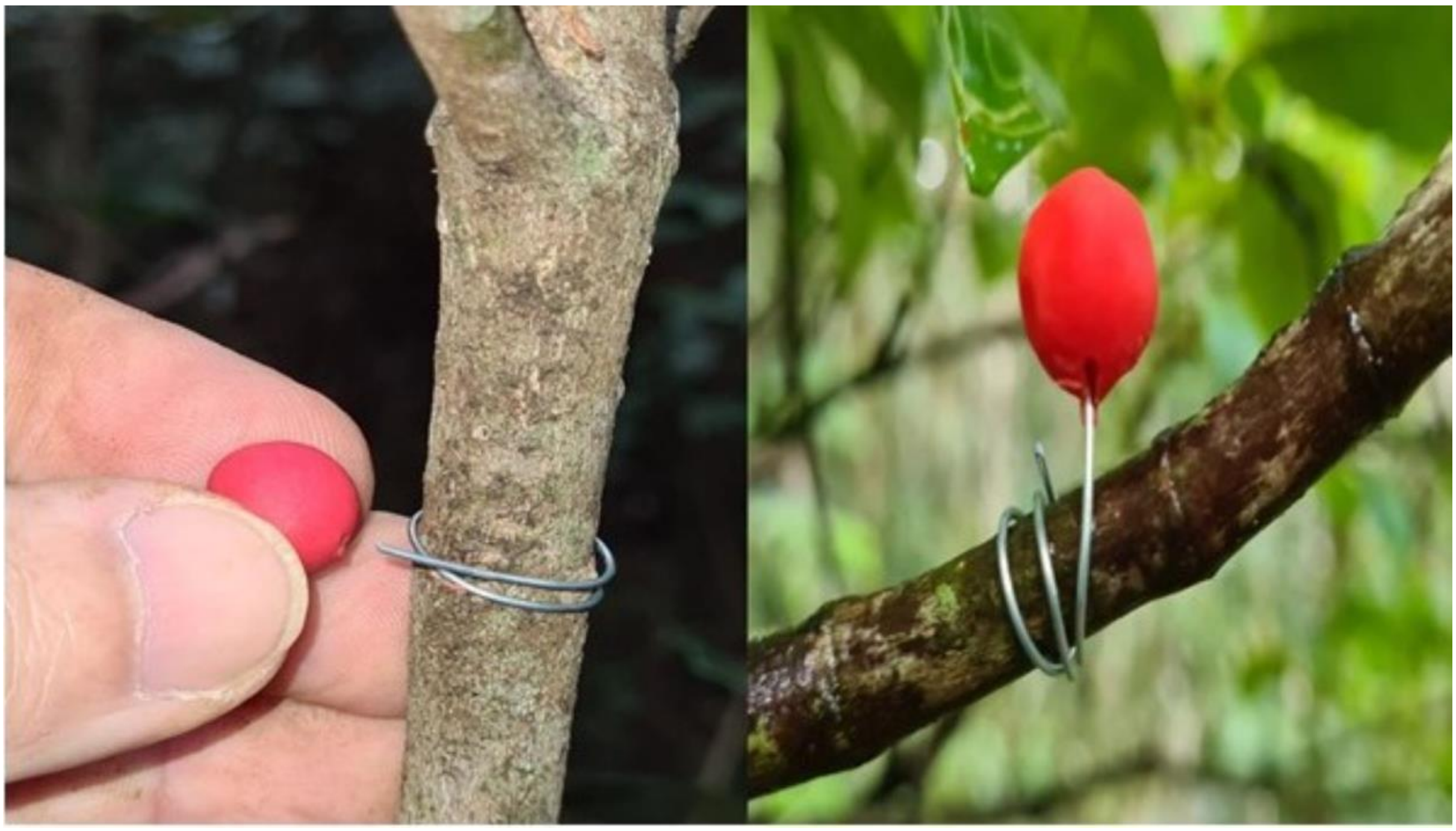

Figure 2. Artificial fruit model preparation and installation. (a) A spherical red plasticine fruit (10 mm diameter) fixed to a thin galvanised wire. (b) The completed fruit model attached to a branch, where it remained exposed for 72 h to record bird peck marks. Photo credit: François Benjamin Vincent Florens (co-author).

3. Selection of experimental plants

Within each forest type, 12 native understorey plants approximately 1.5–2 m tall were selected. Plants were selected according to the following criteria:

- naturally growing native shrubs or small trees;
- no flowers or natural fruits present;
- accessible branches for fruit placement;
- separated by approximately 10–30 m from neighbouring experimental plants;

Note: Different plants were selected during each monthly survey. The canopy openness of each understorey plant was assessed by taking photographs one metre above each understorey plant and analysing them using HabitApp on a smartphone

4. Deployment and exposure period of artificial fruits

Each selected plant received 15 artificial fruits. On each plant, 15 fruit models were attached to branches at about one to two meters above the ground, with inter-fruit spacing of 5-30 cm (Figure 3). Artificial fruits remained in the field for 72 hours.

Note: Passive acoustic monitoring (PAM) was used to identify vocalising bird species in forests undergoing restoration and in invaded forests (Figure 4), simultaneously with the fruit model experiment. Additionally, camera traps can be used to identify which bird species are feeding on artificial fruits.

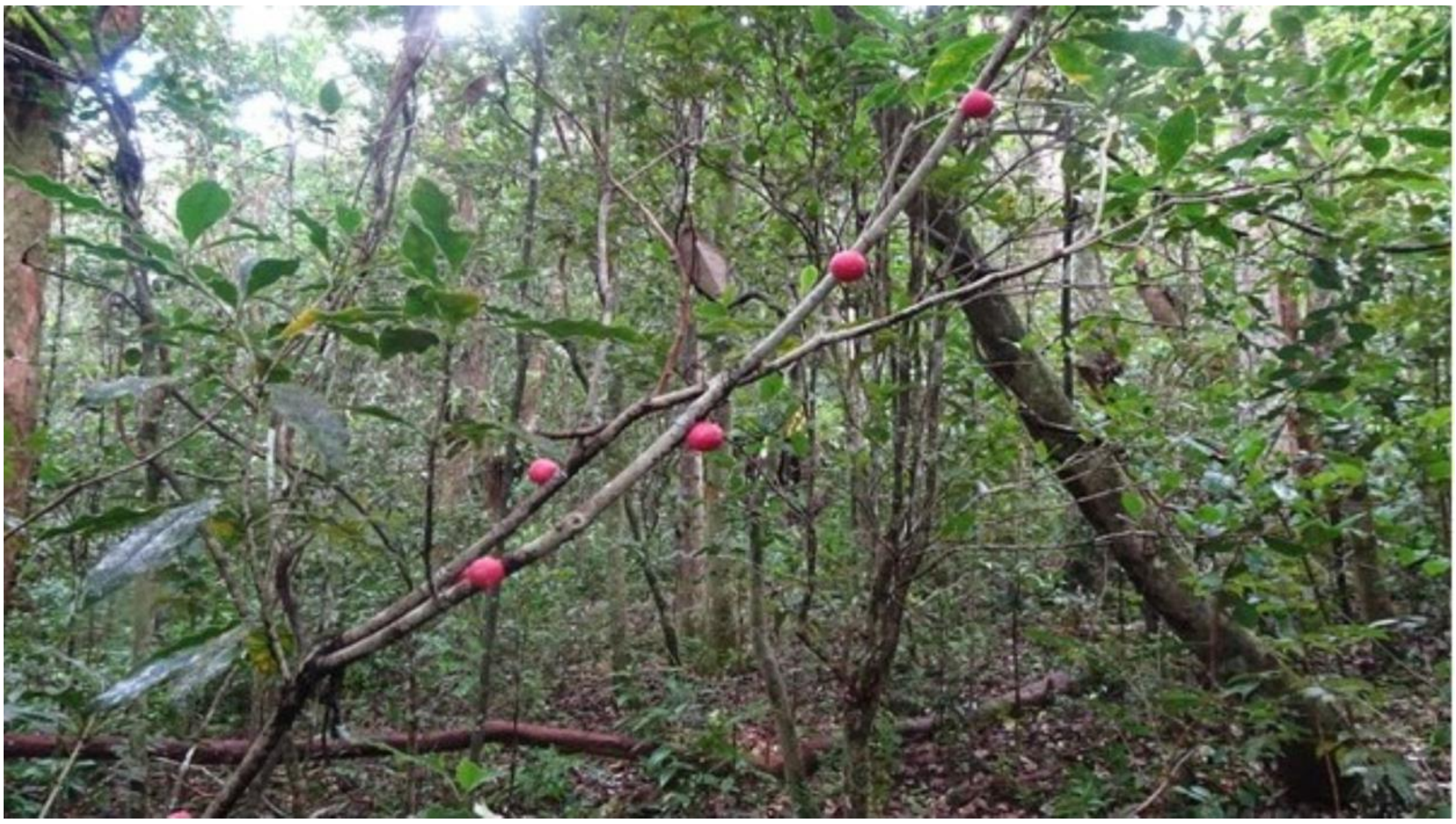

Figure 3. Clay fruit models attached to branches with metal wire.

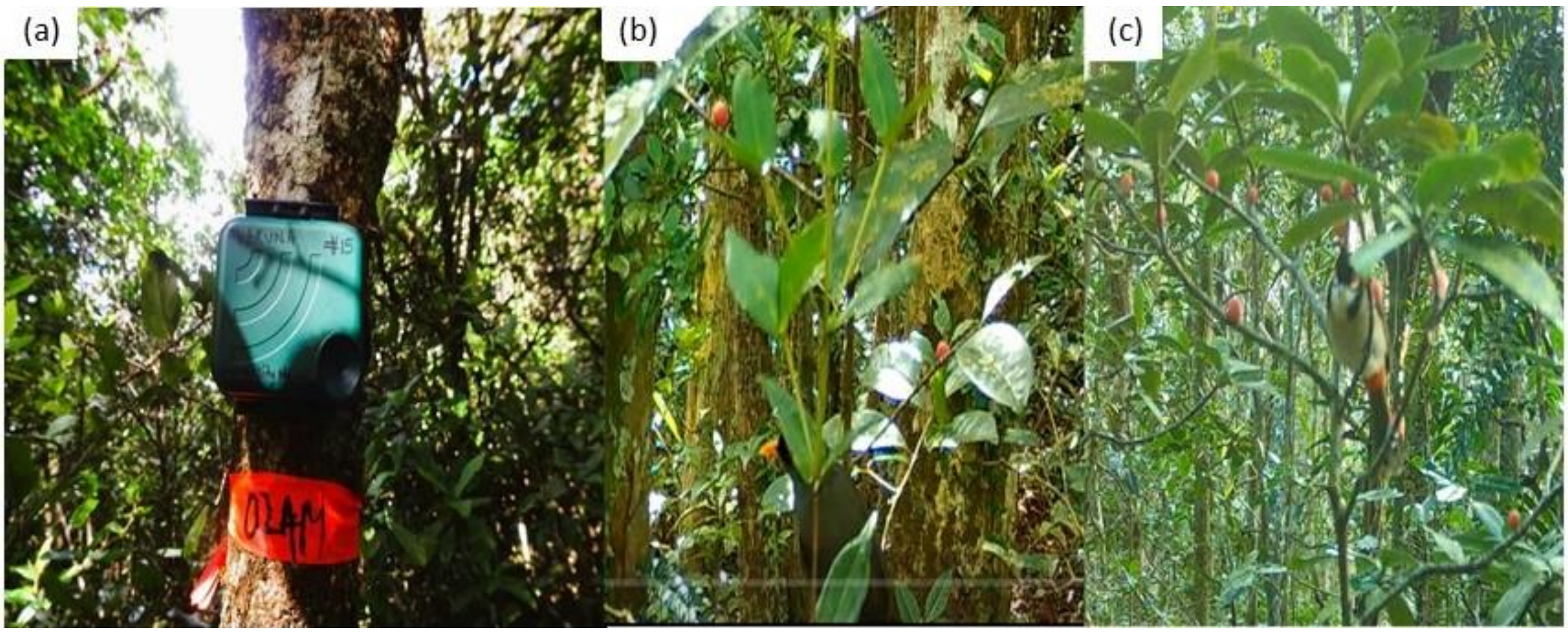

Figure 4. (a) Deployment of an AudioMoth v1.2.0 autonomous recording unit (ARU) on a tree within the study site; (b) Mauritius Bulbul (*Hypsipetes olivaceus*) attacking clay fruit model; (c) Red-whiskered Bulbul (*Pycnonotus jocosus*) attacking clay fruit model. Photo credit: Ghanishta Seeburrun (author)

5. Retrieval of fruits

After 72 hours, all experimental plants were revisited. For each plant, the following were recorded:

- number of intact fruits;
- number of attacked fruits;
- number of missing fruits.

Missing fruits were removed from analysis

6. Identification of attack marks

Each fruit was visually examined immediately after retrieval.

Bird attacks were identified by:

- distinct V-shaped or pointed beak impressions;
- puncture marks consistent with pecking behaviour.

Fruits showing characteristic bird marks were classified as bird attacks.

Fruits displaying mammal tooth marks, arthropod scraping or ambiguous damage were excluded from analyses of avian frugivory (Figure 5).

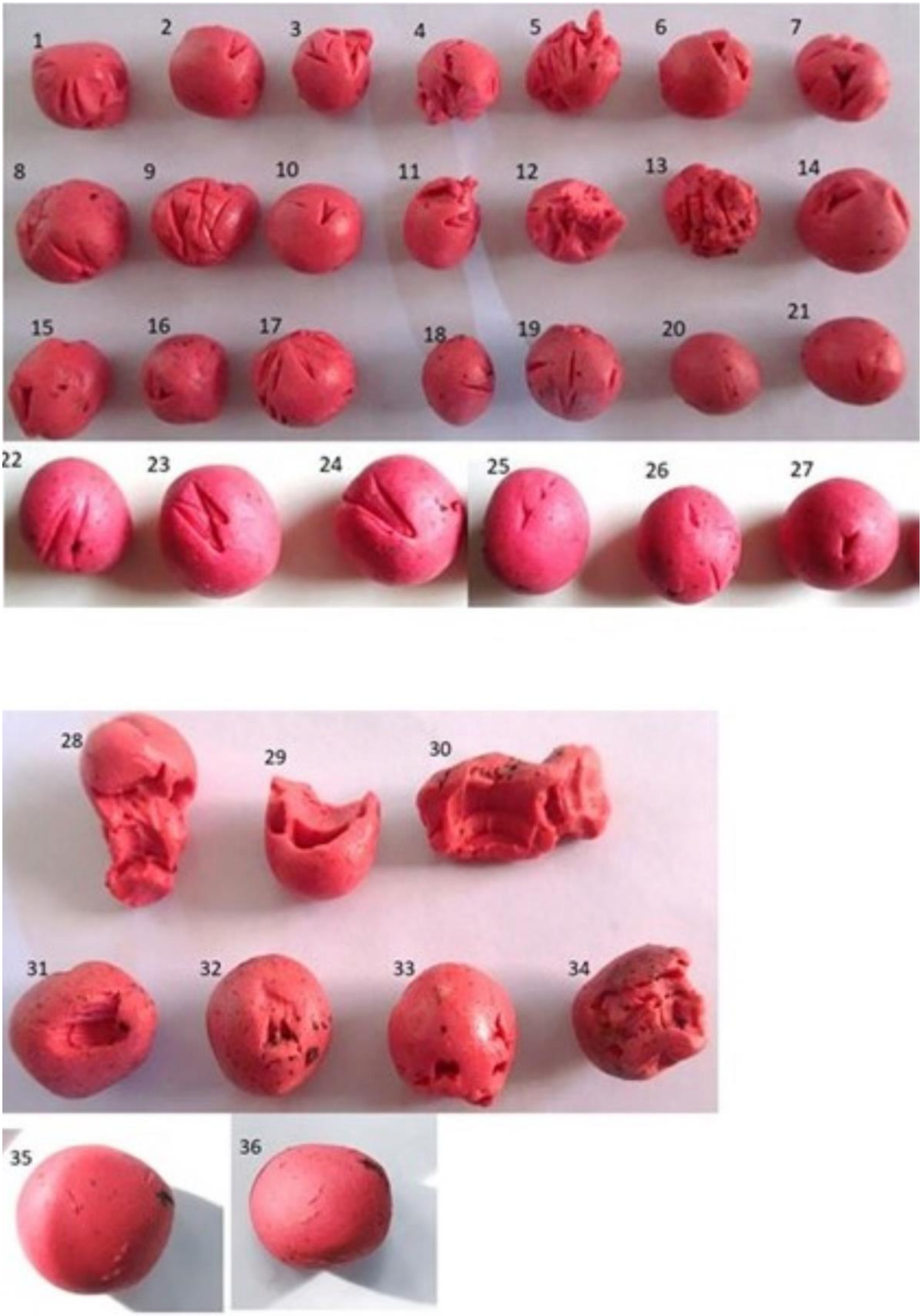

Figure 5. Clay fruit models showing characteristic attack marks by different taxa. Fruit models numbered 1–27 display peck marks consistent with avian frugivory; models 28–30 show bite marks attributed to alien monkeys; models 31–34 show bite marks attributed to alien rats; and

models 35–36 display damage consistent with insect attacks. Photo credit: Ghanishta Seeburrun.

Reference

Alves-Costa CP, Lopes AV. 2001. Using artificial fruits to evaluate fruit selection by birds in the field. *Biotropica*, 33(4), 713–717. DOI:10.1111/j.17447429.2001.tb00230.x.

Balasa NKB, Roquero JO, Lidasan AK, Casim LF, Agduma AR, Tanalgo KC. 2023. Fruit color preference of frugivorous birds in an agroecosystem in southcentral Mindanao, Philippines. *Birds*, 4(2), 190–201. DOI:10.3390/birds4020016.

Ferger SW, Dulle HI, Schleuning M, Böhning-Gaese K. 2016. Frugivore diversity increases frugivory rates along a large elevational gradient. *Oikos*, 125(2), 245–253. DOI:10.1111/oik.02296.

Gagetti BL, Piratelli A, Piña-Rodrigues F. 2016. Fruit color preference by birds and applications to ecological restoration. *Brazilian Journal of Biology*, 76(4), 955–966. DOI: 10.1590/1519-6984.05115.

Galetti M, Traveset A, Jordano P. 2021. Protocol: measuring the macroecological and local effects on plant-animal interaction using artificial fruits. https://www.youtube.com/watch?v=1VOQ_vlt5E8
