# Supplementary material for: Invasive Strawberry Guava weakens avian frugivory in native forests of an oceanic island
Source: PeerJ. 2026 Sep 30;14:e21749. doi: 10.7717/peerj.21749 (PMC13633687; doi:10.7717/peerj.21749)
Supplement: Supplementary file 4 [file peerj-14-21749-s005.docx]

(a)

| **Site** | **Forest type** | **Mean frugivory rate** | **SE** |
| --- | --- | --- | --- |
| Brise Fer | Weeded | 0.154 | 0.022 |
| Brise Fer | Invaded | 0.041 | 0.011 |
| Mare Longue | Weeded | 0.059 | 0.009 |
| Mare Longue | Invaded | 0.022 | 0.004 |

| **Site** | **Paired Wilcoxon signed rank (V)** | **p-value** |
| --- | --- | --- |
| Brise Fer | 66 | 0.0038 |
| Mare Longue | 45 | 0.0092 |

(b)
