# Supplementary material for: Invasive Strawberry Guava weakens avian frugivory in native forests of an oceanic island
Source: PeerJ. 2026 Sep 30;14:e21749. doi: 10.7717/peerj.21749 (PMC13633687; doi:10.7717/peerj.21749)
Supplement: Supplementary file 5 [file peerj-14-21749-s006.docx]

| **Common name** | **Scientific name** | **Origin** | **Diet description** |
| --- | --- | --- | --- |
| Mauritius Bulbul | *Hypsipetes olivaceus* | Endemic | Frugivore: Diet consists mainly of native and some alien fruits. Its diet can also include flowers, nectar, insects, arachnids and endemic geckos (Cheke, 1987; Bissessur, Probst & Florens, 2017; Bissessur et al., 2019; Heinen et al., 2023). |
| Echo Parakeet | *Alexandrinus eques* | Endemic | Frugivore: Diet consists mostly of fruits, flowers, leaves, seeds, twigs, bark and sap (Jones, 1987; Safford & Hawkins, 2013; Heinen et al., 2023). |
| Pink Pigeon | *Nesoenas mayeri* | Endemic | Frugivore: Diet consists of fruits, flowers, leaves, young shoots and seeds and occasionally invertebrates (Jones, 1987; Hume, 2011). |
| Common Myna | *Acridotheres tristis* | Non-native | Frugivore: Diet consists of fruits, insects, seeds, geckos, skink (Sengupta, 1976; Bissessur & Florens, 2018). |
| Madagascar Red Fody | *Foudia madagascariensis* | Non-native | Frugivore: Diet consists mainly of fruits, seeds and insects (Safford & Hawkins, 2013; Nature Seychelles, 2024). |
| Mauritius Cuckooshrike | *Lalage typica* | Endemic | Frugivore: Mostly feeds on insects but occasionally includes fruit in its diet (Cheke, 1987; Safford & Hawkins, 2013). |
| Madagascar Turtle-dove | *Nesoenas cicur* | Non-native | Frugivore: Diet consists of seeds, fruit and insects (Safford & Hawkins, 2013; BirdLife International, 2025). |
| Red-whiskered Bulbul | *Pycnonotus jocosus* | Non-native | Frugivore: Diet consists of fruits, insects and nectar (Linnebjerg et al., 2010; Safford & Hawkins, 2013) |
| Mauritius Grey White-eye | *Zosterops mauritianus* | Native | Diet consists of fruits, insects and nectar (Cheke, 1987; Safford & Hawkins, 2013) |
| Mauritius Kestrel | *Falco punctatus* | Endemic | Carnivore: Diet consists mainly of Phelsuma day geckos but can also include insects, Grey white-eye and small alien mammals like house shrews (Jones, 1987; Carpouron et al., 2023) |
| Mascarene Paradise flycatcher | *Terpsiphone bourbonnensis* | Native | Obligate insectivore: Diet consists mainly of insects. Lepidoptera form an important part of its diet (Cheke, 1987) |
| Zebra dove | *Geopelia striata* | Non-native | Granivore: Feed mostly on seeds and weeds on the ground (Baptista et al., 2020) |

Baptista LF, Trail PW, Horblit HM, Kirwan GM. 2020. Zebra Dove (*Geopelia striata*). In: Billerman SM, Keeney BK, Rodewald PG, Schulenberg TS eds. *Birds of the World*. Cornell Lab of Ornithology,. DOI: 10.2173/bow.zebdov.01.

BirdLife International. 2025.Species factsheet: Madagascar Turtle-dove *Nesoenas picturatus* . (accessed January 1, 2025).

Bissessur P, Bunsy Y, Baider C, Florens FBV. 2019. Non-intrusive systematic study reveals mutualistic interactions between threatened island endemic species and points to more impactful conservation. *Journal for Nature Conservation* 49:108–117. DOI: 10.1016/j.jnc.2019.04.002

Bissessur P, Florens FBV. 2018. Predation of the Mauritius endemic Macchabé Skink, *Gongylomorphus bojerii* fontenayi, by the Common myna, *Acridotheres tristis*. *Bulletin Phaethon* 48:91–93.

Bissessur P, Probst J-M, Florens FV. 2017. Le Merle noir ou Bulbul de Maurice *Hypsipetes olivaceus*. *Bull. Phaethon* 46:86–90.

Carpouron CJ, Zuël N, Monty MLF, Florens FBV. 2023. Breeding success of an endangered island endemic kestrel increases with extent of invasion by an alien plant species. *Journal for Nature Conservation* 72:126366. DOI: 10.1016/j.jnc.2023.126366.

Cheke AS. 1987. The ecology of the smaller land-birds of Mauritius. In: *Studies of Mascarene Island birds*. New York: Cambridge University Press, 151–207.

Heinen JH, Florens FBV, Baider C, Hume JP, Kissling WD, Whittaker RJ, Rahbek C, Borregaard MK. 2023. Novel plant–frugivore network on Mauritius is unlikely to compensate for the extinction of seed dispersers. *Nature Communications* 14:1019. DOI: 10.1038/s41467-023-36669-9.

Hume JP. 2011. Systematics, morphology, and ecology of pigeons and doves (Aves: Columbidae) of the Mascarene Islands, with three new species. *Zootaxa* 3124:1–62. DOI: 10.11646/zootaxa.3124.1.1.

Jones CG. 1987. The larger land-birds of Mauritius. In: *Studies of Mascarene Island Birds*. New York: Cambridge University Press, 208–300.

Linnebjerg J, Hansen DM, Bunbury N, Olesen JM. 2010. Diet composition of the invasive red-whiskered bulbul *Pycnonotus jocosus* in Mauritius. *Journal of tropical ecology* 26:347–350. DOI: 10.1017/S0266467409990617.

Nature Seychelles. 2024.Madagascar Fody. *Available at* *https://natureseychelles.org/seychelles-wildlife/introduced-land-birds/madagascar-fody/* (accessed December 11, 2025).

Safford R, Hawkins F. 2013. *The Birds of Africa: The Malagasy Region: Madagascar, Seychelles, Comoros, Mascarenes*. London: Bloomsbury Publishing Plc.

Sengupta S. 1976. Food and feeding ecology of the common myna, *Acridotheres tristis* (Linn.). 42:338–345.
