# Supplementary material for: Invasive Strawberry Guava weakens avian frugivory in native forests of an oceanic island
Source: PeerJ. 2026 Sep 30;14:e21749. doi: 10.7717/peerj.21749 (PMC13633687; doi:10.7717/peerj.21749)
Supplement: Supplementary file 6 [file peerj-14-21749-s007.docx]

| **Site** | **Source** | **Df** | **Sum of squares** | **R²** | **F** | **p-value** |
| --- | --- | --- | --- | --- | --- | --- |
| Brise Fer | Forest type | 1 | 0.1558 | 0.271 | 5.947 | 0.002 |
|  | Residual | 16 | 0.4191 | 0.729 |  |  |
| Mare Longue | Forest type | 1 | 0.3447 | 0.176 | 3.409 | 0.007 |
|  | Residual | 16 | 1.6180 | 0.824 |  |  |

(b)

| **Site** | **Source** | **Df** | **Mean square** | **F** | **p-value** |
| --- | --- | --- | --- | --- | --- |
| Brise Fer | Forest type | 1 | 0.00053 | 0.169 | 0.699 |
|  | Residuals | 16 | 0.00316 |  |  |
| Mare Longue | Forest type | 1 | 0.00148 | 0.048 | 0.827 |
|  | Residuals | 16 | 0.03067 |  |  |
