# Supplementary material for: Invasive Strawberry Guava weakens avian frugivory in native forests of an oceanic island
Source: PeerJ. 2026 Sep 30;14:e21749. doi: 10.7717/peerj.21749 (PMC13633687; doi:10.7717/peerj.21749)
Supplement: Supplementary file 7 [file peerj-14-21749-s008.docx]

| **Site** | **Species** | **r²** | **p-value** |
| --- | --- | --- | --- |
| Brise Fer | Pink Pigeon | 0.913 | 0.001 |
|  | Mauritius Grey White-eye | 0.679 | 0.001 |
|  | Madagascar Red Fody* | 0.567 | 0.002 |
|  | Echo Parakeet | 0.518 | 0.003 |
|  | Mauritius Bulbul | 0.407 | 0.020 |
| Mare Longue | Red-whiskered Bulbul* | 0.883 | 0.001 |
|  | Echo Parakeet | 0.726 | 0.002 |
|  | Mauritius Grey White-eye | 0.661 | 0.001 |
|  | Mauritius Bulbul | 0.454 | 0.015 |
|  | Pink Pigeon | 0.403 | 0.022 |
|  | Madagascar Red Fody* | 0.361 | 0.050 |

*Represents exotic birds
