# Supplementary material for: Invasive Strawberry Guava weakens avian frugivory in native forests of an oceanic island
Source: PeerJ. 2026 Sep 30;14:e21749. doi: 10.7717/peerj.21749 (PMC13633687; doi:10.7717/peerj.21749)
Supplement: Supplementary file 8 [file peerj-14-21749-s009.docx]

| **Site** | **Forest type** | **Species (common name)** | **Mean detection rate ± SE** | **Adjusted p-value (FDR)** |
| --- | --- | --- | --- | --- |
| **Brise Fer** | Weeded | Red-whiskered bulbul* | 0.90 ± 0.03 | 0.481 |
|  | Invaded |  | 0.87 ± 0.03 |  |
|  | Weeded | Mauritius Grey White-eye | 0.67 ± 0.07 | 0.088 |
|  | Invaded |  | 0.45 ± 0.06 |  |
|  | Weeded | Pink Pigeon | 0.61 ± 0.09 | 0.088 |
|  | Invaded |  | 0.34 ± 0.12 |  |
|  | Weeded | Echo Parakeet | 0.36 ± 0.04 | 0.257 |
|  | Invaded |  | 0.29 ± 0.02 |  |
|  | Weeded | Madagascar Red Fody* | 0.18 ± 0.05 | **0.006** |
|  | Invaded |  | 0.01 ± 0.005 |  |
|  | Weeded | Mauritius Bulbul | 0.12 ± 0.03 | **0.006** |
|  | Invaded |  | 0.01 ± 0.004 |  |
|  | Weeded | Mauritius Cuckooshrike | 0.09 ± 0.04 | 0.610 |
|  | Invaded |  | 0.04 ± 0.01 |  |
|  | Weeded | Common Myna* | 0.08 ± 0.02 | 0.076 |
|  | Invaded |  | 0.16 ± 0.02 |  |
|  | Weeded | Madagascar Turtle-dove* | 0.04 ± 0.01 | 0.050 |
|  | Invaded |  | 0.01 ± 0.005 |  |
| **Mare Longue** | Weeded | Red-whiskered bulbul* | 0.73 ± 0.10 | 0.141 |
|  | Invaded |  | 0.55 ± 0.09 |  |
|  | Weeded | Mauritius Grey White-eye | 0.35 ± 0.07 | 0.076 |
|  | Invaded |  | 0.16 ± 0.04 |  |
|  | Weeded | Echo Parakeet | 0.22 ± 0.04 | 0.076 |
|  | Invaded |  | 0.09 ± 0.03 |  |
|  | Weeded | Pink Pigeon | 0.17 ± 0.06 | 0.076 |
|  | Invaded |  | 0.006 ± 0.006 |  |
|  | Weeded | Madagascar Red Fody* | 0.08 ± 0.03 | 0.088 |
|  | Invaded |  | 0.004 ± 0.002 |  |
|  | Weeded | Mauritius Bulbul | 0.09 ± 0.02 | **0.016** |
|  | Invaded |  | 0.01 ± 0.005 |  |
|  | Weeded | Mauritius Cuckooshrike | 0.02 ± 0.008 | 0.610 |
|  | Invaded |  | 0.02 ± 0.009 |  |
|  | Weeded | Common Myna* | 0.09 ± 0.04 | 0.610 |
|  | Invaded |  | 0.11 ± 0.03 |  |
|  | Weeded | Madagascar Turtle-dove* | 0.02 ± 0.009 | **0.024** |
|  | Invaded |  | 0.00 ± 0.00 |  |
