# Supplementary material for: Invasive Strawberry Guava weakens avian frugivory in native forests of an oceanic island
Source: PeerJ. 2026 Sep 30;14:e21749. doi: 10.7717/peerj.21749 (PMC13633687; doi:10.7717/peerj.21749)
Supplement: Supplementary file 9 [file peerj-14-21749-s010.docx]

| **Acoustic index** | **Spearman’s ρ** | **p-value** |
| --- | --- | --- |
| ACI | 0.686 | < 0.001 |
| ADI | 0.546 | < 0.001 |
| BI | 0.147 | 0.391 |
| NDSI | 0.248 | 0.145 |

**(a)**

(b)

| **Forest type** | **ρ ACI** | **ρ ADI** | **ρ BI** | **ρ NDSI** |
| --- | --- | --- | --- | --- |
| Weeded | 0.626 | 0.654 | 0.059 | 0.042 |
| Invaded | 0.672 | 0.759 | −0.127 | 0.065 |
