# Supplementary material for: Invasive Strawberry Guava weakens avian frugivory in native forests of an oceanic island
Source: PeerJ. 2026 Sep 30;14:e21749. doi: 10.7717/peerj.21749 (PMC13633687; doi:10.7717/peerj.21749)
Supplement: Supplementary file 10 [file peerj-14-21749-s011.docx]

```
 Family: betabinomial  ( logit )
Formula:          cbind(Attacked_fruits, Unattacked_fruits) ~ Forest_type + ACI_z +
    Canopy_z + (1 | patch_month)
Data: dat_ACI

      AIC       BIC    logLik -2*log(L)  df.resid
   1070.4    1094.8    -529.2    1058.4       426

Random effects:

Conditional model:
 Groups      Name        Variance Std.Dev.
 patch_month (Intercept) 0.1002   0.3165
Number of obs: 432, groups:  patch_month, 36

Dispersion parameter for betabinomial family (): 9.28

Conditional model:
                  Estimate Std. Error z value Pr(>|z|)
(Intercept)       -3.55191    0.16922 -20.990  < 2e-16 ***
Forest_typeWeeded  1.21865    0.19937   6.113  9.8e-10 ***
ACI_z              0.27653    0.09537   2.900  0.00374 **
Canopy_z           0.01906    0.07824   0.244  0.80753


m_ACI_full:     Canopy_z + (1 | patch_month), zi=~0, disp=~1
               Df    AIC    BIC  logLik deviance  Chisq Chi Df Pr(>Chisq)
m_ACI_nocanopy  5 1068.4 1088.8 -529.22   1058.4
m_ACI_full      6 1070.4 1094.8 -529.19   1058.4 0.0595      1     0.8073

               df      AIC
m_ACI_nocanopy  5 1068.431
m_ACI_full      6 1070.371

               Df    AIC    BIC  logLik deviance  Chisq Chi Df Pr(>Chisq)
m_base_ACI      4 1073.5 1089.8 -532.78   1065.5
m_ACI_nocanopy  5 1068.4 1088.8 -529.22   1058.4 7.1206      1    0.00762 **
---
               df      AIC
m_base_ACI      4 1073.551
m_ACI_nocanopy  5 1068.431
```
